# Enhancement of the Chiroptical Properties of *o*‑OPE through Arene–Perfluoroarene Interactions

**DOI:** 10.1021/acs.orglett.5c02277

**Published:** 2025-07-30

**Authors:** Darío Otero, Álvaro Martínez-Pinel, Ana M. Ortuño, Luis Álvarez de Cienfuegos, Juan M. Cuerva, Giovanna Longhi, Alba Millán, Delia Miguel

**Affiliations:** † Departamento de Química Orgánica, Unidad de Excelencia de Química (UEQ), C. U. Fuentenueva, 16741 Universidad de Granada, 18071 Granada, Spain; ‡ Department of Molecular and Translational Medicine, Università di Brescia, 25121 Brescia, Italy; § Istituto Nazionale di Ottica (INO), CNR, Research Unit of Brescia, Via Branze 45, 25123 Brescia, Italy; ∥ Departamento de Fisicoquímica, UEQ, Facultad de Farmacia, C. U. Cartuja, Universidad de Granada, 18071 Granada, Spain

## Abstract

In this work, we synthesized and studied fluorinated *o*-oligophenylene ethynylenes (*o*-OPEs). Arene–perfluoroarene interactions promote the folding of the extremes of the OPE stabilizing folded conformations and extending the helical conformation to two complete turns, thus improving their chiroptical responses compared to the nonfluorinated analogue. Dissymmetry factors *g*_abs_ and *g*_lum_ reached values of ∼3 × 10^–2^ on the perfluorinated compound, which represents a 3-fold increase compared to that of the nonfluorinated analogue and is an exceptional value for a small organic molecule.

## Graphical abstract

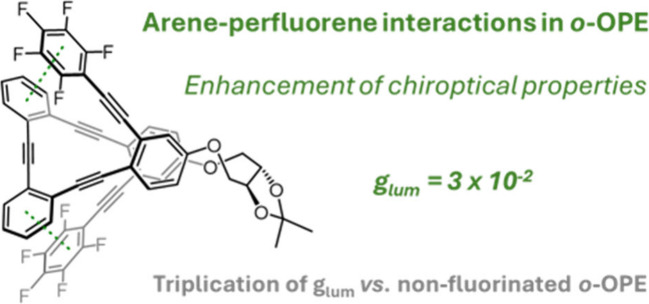

The development of CPL-emitting compounds has experienced exponential growth during the past decade,^1^ due to the promising applications they have in fields as diverse as asymmetric synthesis,^2–6^ chiroptical sensing,^7–9^ chiral optoelectronics,^10^ information sciences, and anticounterfeiting devices.^11–13^ The quantification parameter of CPL is the luminescence dissymmetry factor (*g*_lum_), which is determined by the equation *g*_lum_ = 2 × (*I*_L_ – *I*_R_)/(*I*_L_ + *I*_R_), where *I*_L_ and *I*_R_ are defined as the intensity of left- and right-handed circularly polarized light, respectively. To date, many strategies have been developed to improve the *g*_lum_ value, including supramolecular assembly of chiral materials^14,15^ and the optimization of simple organic molecules,^16–18^ among others.

Concerning this last approach, during the past several years, our group has been involved in the optimization of the chiroptical properties of helical scaffolds based on the *o*-oligophenylene ethynylene (*o*-OPE) skeleton.^19–22^ In this sense, we described chiral stapling as an efficient way to induce a fixed helical sense in the structure, allowing the preparation of ratiometric probes in the ground and excited states.^23^ With this strategy, we have prepared systems with up to four complete turns,^20^ where a linear relationship between the magnetic dipole transition moment and the length of the helix was observed. However, dissymmetry factor *g*_lum_ did not follow a trend, suggesting that the helical chirality was lost when the distance to the chiral nucleus increased due to the flexibility of the structures. Although coordination with carbophilic metals as Ag­(I) favors folding, this effect is partial and involves a substantial quenching of the fluorescence, reducing the applications based on CPL. To overcome this issue, in this work we aim to introduce substituents that promote an enhancement of noncovalent interactions. This fact would facilitate the folding of the structure without the need to use metals. Within this context, aromatic and perfluoroaromatic compounds present opposite quadrupoles derived from the large difference in electronegativity between the hydrogen and fluorine atoms, thus favoring an interaction between them due to an enthalpically favorable stacking,^24–26^ which can also be affected by Pauli repulsion and dispersion interactions.^24,25,27^ The strength of these arene–perfluoroarene π–π interactions has been proved in the oligomer conformation of *p*-phenylene ethynylene derivatives^28^ and in the folding of macrocycles based on *o-*phenylenes.^29^ In addition, these interactions have been recently exploited to promote peptide folding by force-driven clamping^30^ and two-dimensional peptide assembly,^31^ to hierarchically build supramolecular chirality,^32^ to induce chiroptical inversion and precise ee detection of chiral acids,^33^ and to promote CPL switching^34^ and amplification,^35^ among others.^36^

Herein, we have designed three fluorinated compounds, (*S*,*S*,*P*)-**1-F**_**1**_, (*S*,*S,P*)-**1-F**_**3**_, and (*S*,*S*,*P*)-**1-F**_**5**_, that are two-turn helix analogues to our previously described nonfluorinated (*S*,*S,P*)-**1**.^20^ The fluorine atoms have been introduced into the benzene rings at both ends of the helix (Figure 1). Our working hypothesis states that the inclusion of fluorine atoms would improve the folding of the structure with respect to (*S*,*S,P*)-**1** (73.6% folded as shown by DFT calculations) and consequently the *g*_lum_ value. This increase in the chiroptical response, together with the preservation of the fluorescent quantum yield, would afford an improved candidate for CPL applications.

**Figure 1:**
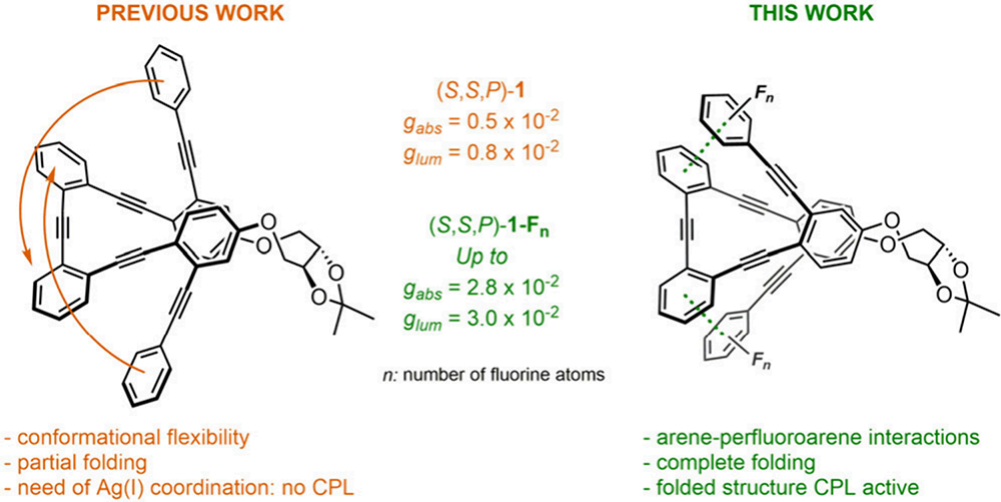
Working hypothesis and structure of enantiopure (*S*,*S*,*P*)-**1**-**F**_***n***_.

The synthesis of enantiopure compounds (*S*,*S,P*)-**1-F**_***n***_ (*n* = 1, 3, or 5) was carried out following our previously described methodology.^20^ Thus, Sonogashira cross-coupling reactions between enantiopure brominated core (*S*,*S*,*P*)-**I** and corresponding alkynes **II-F**_***n***_ afforded the final products in good yields (Scheme 1). Likewise, enantiomers (*R*,*R,M*)-**1-F**_***n***_ were prepared from (*R*,*R*,*M*)-**I**.

**Scheme 1:**
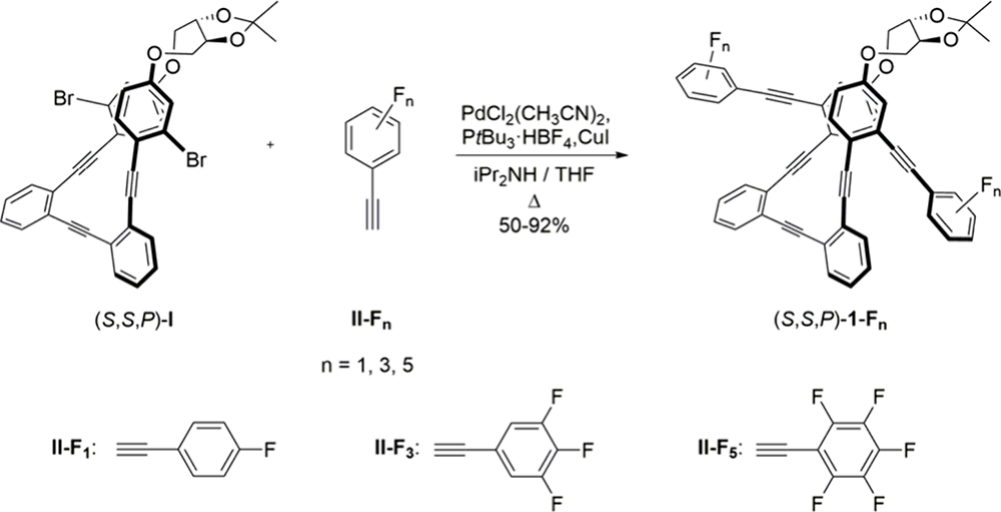
Synthesis of (*S*,*S*,*P*)-**1**-**F**_***n***_

With the three pairs of enantiomers in hand, we evaluated their photophysical and chiroptical properties. First, we studied the photophysical properties of (*S*,*S*,*P*) enantiomers in CH_2_Cl_2_ solutions (Figure 2, left panels). All three compounds, (*S*,*S*,*P*)-**1-F**_**1**_, (*S*,*S*,*P*)-**1-F**_**3**_, and (*S*,*S*,*P*)-**1-F**_**5**_, presented similar absorbance and emission spectra. The compounds presented absorbance maxima at 274, 282, and 279 nm, respectively, with a shoulder centered at 340 nm in all cases (Figure 2, central panels). Fluorescence spectra showed a single peak with a maximum at ca. 423 nm with similar values of fluorescence quantum yield for the three derivatives (30%, 20%, and 21%, respectively), which were in the range of, or higher than, that observed for (*S*,*S*,*P*)-**1** (19%).^20^ To shed light on the effect of fluorine atoms on the electronic characteristics of the OPEs, we explored the influence of the solvent nature on the photophysical properties of (*S*,*S*,*P*)-**1-F**_**5**_. Both the absorbance and the emission were comparable in different solvents (Figure S10), ranging from apolar (hexane) to polar ones, including protic (MeOH) and aprotic (MeCN) solvents. Fluorescence decay curves fitted to a biexponential function in all cases (Table S1), the average lifetime being around 4 ns in all of the solvents.^20^ Concerning fluorescence quantum yields (QYs), small differences were observed, the highest value being in CH_2_Cl_2_ (21%) and the lowest in MeOH (16%) (Table S1).^20^ The behavior of mono- and trifluorinated derivatives was also analyzed in these edge cases. In both solvents, a decrease in the QY was observed with an increase in the number of fluorine substituents, but there were no significant changes in the fluorescence lifetime values. On the other hand, as for the case of (*S*,*S,P*)-**1-F**_**5**_, similar differences were observed for each compound between solvents. This suggests that these properties are independent of the environment, thus supporting structural robustness.

**Figure 2:**
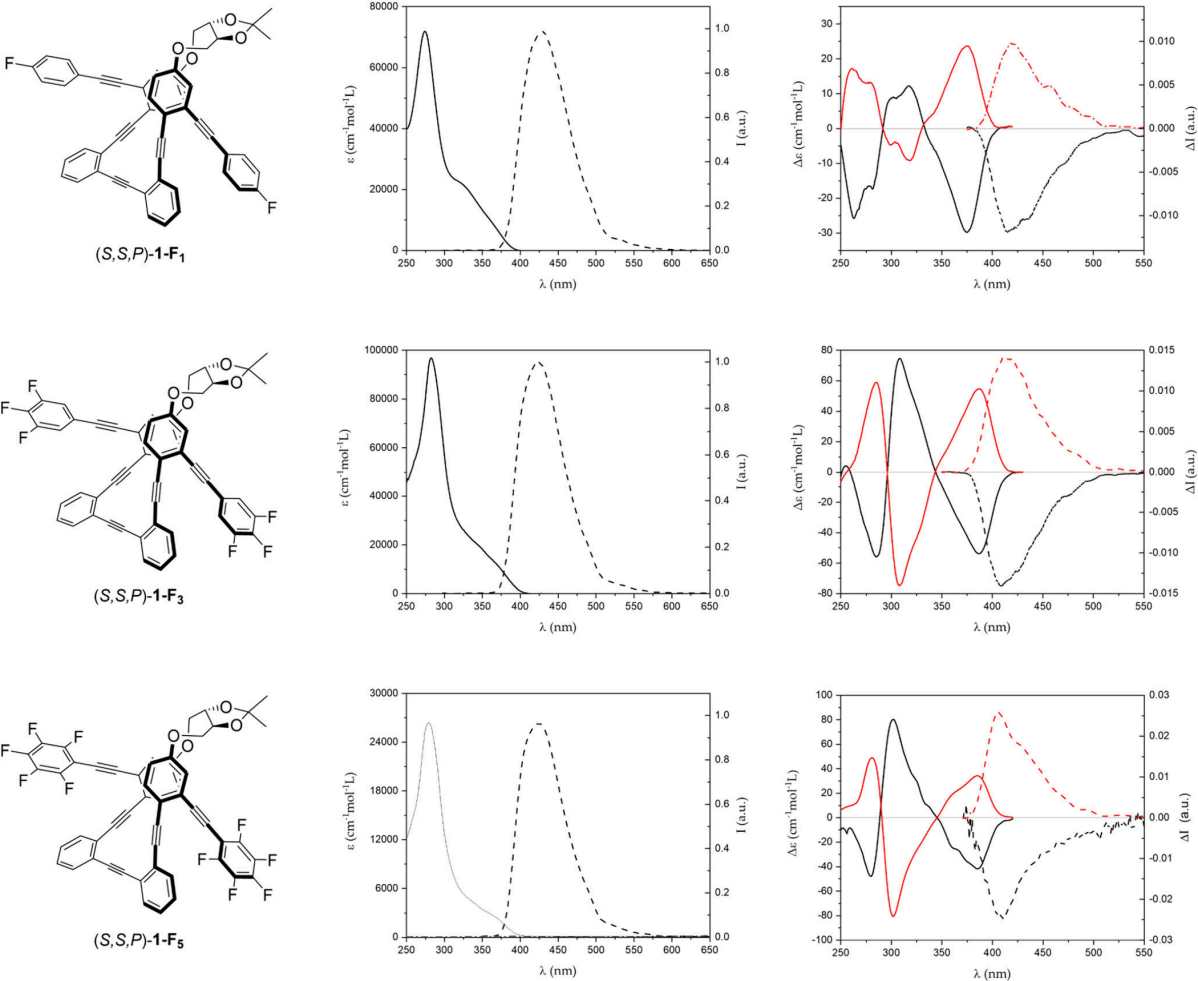
Structures (left) of (*S*,*S*,*P*)-**1**-**F**_**1**_ (top), (*S*,*S*,*P*)-**1**-**F**_**3**_ (middle), and (*S*,*S*,*P*)-**1**-**F**_**5**_ (bottom). UV–vis (solid line) and fluorescence (dashed line) spectra (center) of (*S*,*S*,*P*)-**1**-**F**_***n***_. ECD (solid line) and CPL (dashed line) (right) of (*S*,*S*,*P*)-**1**-**F**_***n***_ (red line) and (*R*,*R*,*M*)-**1**-**F**_***n***_ (black line). All of the spectra were recorded using 1.5 × 10^–5^ M solutions in CH_2_Cl_2_.

Next, we investigated the chiroptical responses of the three enantiomeric pairs (*S,S,P*)/(*R,R,M*)-**1-F**_**1**_, (*S,S,P*)/(*R,R,M*)-**1-F**_**3**,_ and (*S,S,P*)/(*R,R,M*)-**1-F**_**5**_ in CH_2_Cl_2_ solutions (Figure 2, right). The similarities in the ECD spectra of (*S*,*S*,*P*)-**F**_***n***_ compared to those obtained for nonfluorinated (*S*,*S*,*P*)-**1** ^20^ (Figure S11a) revealed that the previously observed *P* helicity is maintained. For the first transition, an increase in the maximum ECD signal together with a bathochromic shift was observed from (*S*,*S*,*P*)-**1-F**_**1**_ (Δε = 23.7 M^–1^ cm^–1^ at 375 nm) to (*S*,*S*,*P*)-**1-F**_**3**_ (Δε = 54.7 M^–1^ cm^–1^ at 387 nm). For (*S*,*S*,*P*)-**1-F**_**5**_, the wavelength of the maxima was quite similar to that of (*S*,*S*,*P*)-**1-F**_**3**_, but a lower ECD value was obtained (Δε = 30.4 M^–1^ cm^–1^ at 385 nm), which was consistent with the lower values of molar absorptivity. Regarding CPL spectra, a direct relationship between the number of fluorine atoms and the CPL signal was observed, reaching a maximum value for the perfluorinated derivative.

In addition, we measured the ECD and CPL of (*S*,*S,P*)-**1-F**_**5**_ and (*R*,*R,M*)-**1-F**_**5**_ solutions in the previously mentioned solvents. Concerning ECD, small differences were observed, affording slightly higher intensities in the most polar solvents (Figure S13a). Thus, in a MeCN solution, the less energetic band, which displayed a positive Cotton effect, presents a Δε value of 34.3 M^–1^ cm^–1^ at 385 nm. The comparison of the emission properties led us to similar conclusions. As one can see in the CPL spectra (Figure S13b), both the shape and the intensity were almost identical, showing no significant influence of the solvent on the excited state of the molecule.

Finally, to shed light on the impact of the fluorine atoms on chiroptical properties, we measured the absorption and luminescence dissymmetry factors (*g*_abs_ and *g*_lum_, respectively) of all (*S*,*S*,*P*) enantiomers. As one can see in Table 1, absolute values increased with the number of fluorine atoms compared to those of nonfluorinated derivative (*S*,*S*,*P*)-**1**.^20^ Specifically, the introduction of one ((*S*,*S*,*P*)-**1**-**F**_**1**_) and three ((*S*,*S*,*P*)-**1**-**F**_**3**_)) fluorine atoms into the outermost benzene rings led to 13% and 140% increases in *g*_abs_ and 12.5% and 75% increases in *g*_lum_, respectively. This enhancement is much more prominent in (*S*,*S*,*P*)-**1**-**F**_**5**_, where arene–perfluoroarene interactions are maximized by the introduction of five fluorine atoms. In this case, the chiroptical response is boosted to nearly 4 times its original value. For this compound_,_ we also obtained very similar *g*_lum_ values in all of the solvents (see the Supporting Information), suggesting the stability of the structure is independent of the environment.

**Table 1:** *g*_abs_ and *g*_lum_ Values of Fluorinated *o*-OPEs in CH_2_Cl_2_

compound	*g*_abs_ (λ (nm))	*g*_lum_ (λ (nm))
(*S,S,P*)-**1**	0.45 × 10^–2^ (387)	0.8 × 10^–2^ (402)
(*S,S,P*)-**1-F**_**1**_	0.51 × 10^–2^ (376)	0.9 × 10^–2^ (406)
(*S,S,P*)-**1-F**_**3**_	1.2 × 10^–2^ (387)	1.4 × 10^–2^ (400)
(*S,S,P*)-**1-F**_**5**_	2.8 × 10^–2^ (389)	3 × 10^–2^ (398)

This fact highlights the strength of the π–π interactions between the arene and perfluoroarene rings when five fluorine atoms are present. It is noteworthy that the *g*_abs_ magnitude increases more than 5-fold with respect to (*S*,*S*,*P*)-**1** and is even better than that obtained in the folded (*S*,*S*,*P*)-**1**:Ag­(I) complex (2.5 × 10^–2^).^20^ This demonstrates that the structure is completely folded in solution. To provide further evidence, we carried out ECD titrations of compounds (*S*,*S*,*P*)-**1**-**F**_***n***_, adding increasing quantities of Ag­(I) without changing the ligand concentration. As depicted in Figure S19, the behavior of extreme cases (*S*,*S*,*P*)-**1** and (*S*,*S*,*P*)-**1**-**F**_**5**_ is quite different. For (*S*,*S*,*P*)-**1**-**F**_**5**_, no significant change in the last band after the metal addition was obtained, demonstrating again that the folding is not dependent on the coordination. For mono- and trifluorinated derivatives, we observed an intermediate evolution of the ECD signal.

To rationalize observed dissymmetry ratios *g*_abs_ and *g*_lum_ and their increase upon fluorination, DFT calculations have been performed. One must be aware, however, of the difficulties for such approach in adequately representing arene–perfluoroarene π–π interactions, where the interplay of Pauli repulsion, dispersion interactions, and electrostatics has a role. We considered in detail the two cases (*S*,*S*,*P*)-**1** and (*S*,*S*,*P*)-**1-F**_**5**_, performed a conformational search, and optimized the structures (Supporting Information). In both cases, a folded structure, stabilized by π–π interactions, is by far the lowest-energy one (conformer “a” (Tables S2 and S3)). Conformers are described by the reciprocal orientation of the phenylene ethynylene moieties (τ_1_–τ_3_ angles) and by conformational degrees of freedom of the chiral staple, which are responsible for the stabilization of the (*S*,*S*,*P*) form. At the adopted level of calculations, the first “unfolded” structure found for (*S*,*S*,*P*)-**1-F**_**5**_ (conformer “f” (Table S2)) is 5.12 kcal/mol higher in energy above the most stable one, while the first “unfolded” structure found for (*S*,*S*,*P*)-**1** (conformer “f”, Table S3) is only 0.74 kcal/mol higher than the lowest-energy structure. It should be noted that while the unfolded structure is not populated at all in (*S*,*S*,*P*)-**1-F**_**5**_, it represents 21% for (*S*,*S*,*P*)-**1**, being the second one in energy over the folded one. With the aim of comparing folded and unfolded geometries, we calculated the difference in energy also for (*S*,*S*,*P*)-**1-F**_**1**_ (1.5 kcal/mol) and (*S*,*S*,*P*)-**1-F**_**3**_ (2.66 kcal/mol). It appears that fluorine atoms help in stabilizing the folded structure, as expected. Also, the trend observed on the calculated distance between terminal arenes progressively decreases with an increase in the number of fluorine atoms (Figure S21 and Table S5). Comparing calculated CD spectra, the most stable conformer does not show significant differences among the four examined compounds, attributing the observed spectroscopic differences to varying degrees of conformational disorder. The calculated CD spectrum of the first unfolded conformer shows in all cases a positive, weak, lowest-energy band, while the main differences are observed at 300 nm with a low-intensity band of opposite sign with respect to the stable folded structure. These theoretical results help to explain why the solvent dependence of CD signals is observed particularly in this spectroscopic region for compound (*S*,*S*,*P*)-**1** (Figure S12),^20^ which lacks the stabilization effect of fluorine atoms, and not for (*S*,*S*,*P*)-**1-F**_**5**_ (Figure S13a). Furthermore, the unfolded structure presents a higher absorbance in the 350–400 nm region that contributes to giving a lower *g*_abs_ value for (*S*,*S*,*P*)-**1** with respect to (*S*,*S*,*P*)-**1-F**_**5**_ (Figure S25).

Finally, the CPL brightness (*B*_CPL_) has been defined as an important parameter in chiroptical materials, as it includes information relative to both chirality and luminescence.^37^ For (*S*,*S*,*P*)-**1**-**F**_**5**_, *B*_CPL_ reaches 82 M^–1^ cm^–1^, making this scaffold an outstanding candidate for chiroptical applications.

In conclusion, we synthesized three enantiomeric pairs of *o*-OPEs with an increasing number of fluorine atoms. Although they presented almost identical photophysical properties, striking differences were observed in their chiroptical responses. The introduction of one ((*S*,*S,P*)-**1-F**_**1**_), three ((*S*,*S,P*)-**1-F**_**3**_), and five ((*S*,*S,P*)-**1-F**_**5**_) fluorine atoms into both outermost benzene rings led to 1.12-, 1.75-, and almost 4-fold increases, respectively, in *g*_lum_. This enhancement is related to the stabilization of the helical arrangement by greater arene–perfluorarene interactions taking place with an increasing number of F atoms. The exceptional magnitude of *g*_lum_ found for (*S*,*S*,*P*)-**1**-**F**_**5**_ together with the adequate value of quantum yield makes this simple organic molecule a promising scaffold as a model for CPL applications.

## Supplementary Material



## Data Availability

The data underlying this study are available in the published article and its Supporting Information.

## Notes

Synthetic procedures and characterization of compounds, copies of NMR spectra, additional ECD and CPL spectra, lifetimes and QYs, theoretical calculation detailss and conformer analyses (PDF)
