# Supplementary material for: Enhancement of the Chiroptical Properties of o‑OPE through Arene–Perfluoroarene Interactions
Source: Org Lett. 2025 Jul 30;27(31):8459–63. doi: 10.1021/acs.orglett.5c02277 (PMC12340963; doi:10.1021/acs.orglett.5c02277)
Supplement: Supplementary file 1 [file ol5c02277_si_001.pdf]

SUPPORTING INFORMATION

Enhancement of Chiroptical Properties of *o*-OPE through Arene-Perfluoroarene Interactions

Dario Otero,[a]‡ Álvaro Martínez-Pinel,[a]‡ Ana M. Ortuño,[a] Luis Álvarez de Cienfuegos,[a] Juan M. Cuerva,[a] Giovanna Longhi,[b,c] Alba Millán,[*a] and Delia Miguel [*d]

[a] Departamento de Química Orgánica, Unidad de Excelencia de Química (UEQ), C. U. Fuentenueva, Universidad de Granada, 18071, Granada, Spain

[b] Department of Molecular and Translational Medicine, Università di Brescia, Brescia, Italy

[c] Istituto Nazionale di Ottica (INO), CNR, Research Unit of Brescia, Via Branze 45, 25123 Brescia, Italy

[d] Departamento de Fisicoquímica, UEQ, Facultad de Farmacia, C. U. Cartuja, Universidad de Granada, 18071, Granada, Spain

Table of contents

1. GENERAL INFORMATION 2
2. SYNTHETIC DETAILS 2
 2.1. Synthesis of Alkyne II-F_5 2
 2.2. General Procedure for the Synthesis of *(R,R,M)*- and *(S,S,P)*-I-F_n and Characterisation 4
3. NMR SPECTRA OF NEW COMPOUNDS 6
4. PHOTOPHYSICAL STUDIES 12
 4.1. Absorbance and Emission Spectra of (*S*,*S*,*P*)-1-F_5 in Different Solvents 12
 4.2. Lifetimes and Quantum Yields of (*S*,*S*,*P*)-1-F_n 12
 4.3. CD and CPL Spectra 14
 4.4. Photophysical measurements for 1-F_1 and 1-F_3 in MeOH 15
 4.5. Luminescence Dissymmetry Factor (g_{lum}) Graphics 16
 4.6. ECD Titration of (*S*,*S*,*P*)-1 and (*S*,*S*,*P*)-1-F_n with Ag(I) 17
5. THEORETICAL CALCULATIONS 18

5.1. Conformers' optimization 18
5.2. Calculated spectra 22
5.3. Coordinates for the lowest energy structures of compounds (*S*,*S*,*P*)-1-F_n 26
5.4. Coordinates of the first (in energy) partially folded structures of (*S*,*S*,*P*)-1-F_n... 34
6. REFERENCES... 42

1. GENERAL INFORMATION

Unless otherwise stated, all reagents and solvents were purchased from commercial sources and used without further purification. Anhydrous THF was freshly distilled over Na/benzophenone. Compounds (*S*,*S*,*P*)-**I** and (*R*,*R*,*M*)-**I** were synthesized following the reported procedure.[S1] Alkynes **II**-F_1 and **II**-F_3 were purchase from BLDpharm. Flash column chromatography was carried out using silica gel 60 (230-400 mesh) as the stationary phase. All mixed solvent eluents are reported as v/v solutions. Analytical TLC was performed on aluminium sheets coated with silica gel with fluorescent indicator UV_{254} (Alugram SIL G/UV_{254}, Mackerey-Nagel, Germany) and observed under UV light (254 nm) and/or stained with phosphomolybdic acid (5% ethanol solution). All ^{1}H, ^{19}F and ^{13}C NMR spectra were recorded on Bruker Avance Neo (400 MHz or 500 MHz) spectrometers at a constant temperature of 298 K. Chemical shifts are reported in ppm and referenced to residual solvent. Coupling constants (*J*) are reported in Hertz (Hz). Multiplicities are abbreviated as follow: s = singlet, d = doublet, t =triplet m = multiplet, dd = doublet of doublets, td = triplet of doublets, ddd = doublet of doublet of doublets, dt = doublet of triplets. Proton assignment was carried out by 2D NMR experiments (COSY, HSQC and/or HMBC). Assignment of the ^{13}C NMR multiplicities was accomplished by DEPT techniques. ESI-TOF mass spectra were recorded in a Waters Xevo G2-XS QTof spectrometer. Both absorption and emission measurements were performed in an Olis DSM172 spectrophotometer using a 1.0 cm path-length quartz cell and 1.5×10^{-5} M solutions of the compounds in HPLC grade solvents. A xenon lamp of 150 W was used for measurements. Excitation wavelength was 278 nm.

2. SYNTHETIC DETAILS

2.1. Synthesis of Alkyne II-F_5

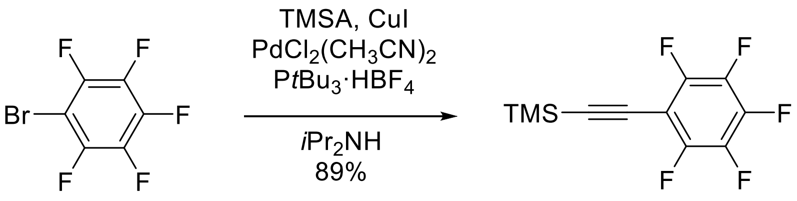

S1

A solution of TMSA (873 mg, 8.9 mmol, 1.1 equiv.) dissolved in the minimum volume of THF was added dropwise to a carefully degassed solution of $Pd(CH_3CN)_2Cl_2$ (104 mg, 0.45 mmol, 0.05 equiv.), P*t*Bu_3·HBF_4 (232 mg, 0.89 mmol, 0.1 equiv.), CuI (76 mg, 0.45 mmol, 0.05 equiv.) and bromopentafluorobenzene (2.0 g, 8.10 mmol, 1.0 equiv.) in *i*Pr_2NH (10 mL). The reaction was stirred for 5 h at room temperature under an argon atmosphere. The mixture was then diluted with pentane (2×20 mL) and washed with saturated aqueous NH_4Cl solution (2×20mL). The organic phase was dried over anhydrous Na_2SO_4, filtered and the solvent was removed under reduced pressure. The residue was purified by flash chromatography (SiO_2, pentane) to give **S1** (1.9 g, 7.9 mmol, 89%) as a yellowish oil. Spectroscopic data matched with those previously reported.[S2] **^{1}H NMR (400 MHz, $CDCl_3$)** δ 0.19 (s, 9H). **^{13}C NMR (101 MHz, $CDCl_3$)** δ 88.13 (C), 86.11 (C), -0.36 (CH_3). **^{19}F NMR (376 MHz, $CDCl_3$)** δ -132.00 – -132.15 (m, 2F), -153.88 (t, *J* = 20.8 Hz, F), -159.81 – -160.03 (m, 2F).

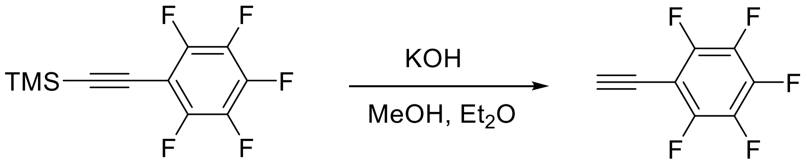

S1 **II-F_5**

The deprotection of **S1** was carried out following the protocol established by Moore *et al.*[S3] An aqueous solution of KOH (0.5 mL, 2g/mL) was added in one portion to a solution of **S1** (557 mg, 2.1 mmol, 1 equiv.) in a mixture of Et_2O (10 mL) and MeOH (15 mL). After being stirred for 1.5 min at room temperature, it was diluted quickly with water (50 mL). The mixture was extracted with pentane and the organic layer was dried over anhydrous Na_2SO_4 and filtered off. The solvent was removed under reduced pressure at 0 °C to give **II-F_5** as a yellowish oil. This crude mixture was used in the next step without further purification.

2.2. General Procedure for the Synthesis of *(R,R,M)*- and *(S,S,P)*-I-F_n and Characterisation

(*R*,*R*,*M*)- or (*S*,*S*,*P*)-**I** (90 mg, 0.13 mmol, 1 equiv.), $PdCl_2(CH_3CN)_2$ (10 mg, 0.039 mmol, 0.3 equiv.), P*t*Bu_3·HBF_4 (20 mg, 0.069 mmol, 0.5 equiv.) and CuI (10 mg, 0.053 mmol, 0.4 equiv.) were placed into a pressure tube and deoxygenated. The solids were dissolved in dry and deoxygenated THF:Et_3N mixture (4:3, 7 mL), the corresponding alkyne **II-F_n** (20 equiv.) was added, and the reaction mixture was heated at 40°C (oil bath) for 20 h. After this time, the reaction was cooled down to room temperature, diluted with EtOAc and washed with brine. The organic phase was dried with Na_2SO_4, filtered, and concentrated in vacuo. Column chromatography of the residue on silica gel (Hexane:EtOAc, 8:2) provided the products (*R*,*R*,*M*)- or (*S*,*S*,*P*)-**I-F_n** as brown oils ((*S*,*S*,*P*)-**1-F_1**: 89 mg, 0.12 mmol, 89 % yield; (*R*,*R*,*M*)-**1-F_1**: 81 mg, 0.10 mmol, 81 % yield; (*S*,*S*,*P*)-**1-F_3**: 101 mg, 0.12 mmol, 92 % yield; (*R*,*R*,*M*)-**1-F_3**: 94 mg, 0.11 mmol, 86 % yield; (*S*,*S*,*P*)-**1-F_5**: 60 mg, 0.06 mmol, 50 % yield; (*R*,*R*,*M*)-**1-F_5**: 15 mg, 0.016 mmol, 12 % yield). Full characterization of (*S*,*S*,*P*)-**1-F_n** enantiomers is shown below. Spectroscopic data of the corresponding (*R*,*R*,*M*)-**1-F_n** compounds matched with those of their corresponding (*S*,*S*,*P*) enantiomers.

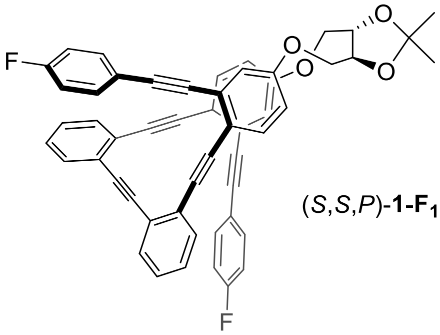

(*S*,*S*,*P*)-**1-F_1**

^{1}H NMR (400 MHz, $CDCl_3$) δ 7.49 (dd, *J* = 7.9, 1.3 Hz, 2H), 7.38 – 7.32 (m, 6H), 7.22 – 7.17 (m, 4H), 7.13 (td, *J* = 7.6, 1.5 Hz, 2H), 6.71 – 6.61 (m, 6H), 6.36 (dd, *J* = 8.6, 2.7 Hz, 2H), 4.34 – 4.26 (m, 4H), 4.27 – 4.16 (m, 2H), 1.44 (s, 6H). **^{13}C NMR (101 MHz, $CDCl_3$)** δ 162.3 (d, $^1J_{C-F}$ = 249.4 Hz, C), 157.3 (C), 133.8 (d, $^3J_{C-F}$ =8.3 Hz, CH), 133.7 (CH), 133.6 (CH), 133.1 (CH), 127.9 (CH), 127.6 (CH), 126.8 (C), 125.3 (C), 124.8 (C), 119.4 (d, $^4J_{C-F}$ = 3.5 Hz, C), 119.1 (C), 116.8 (CH), 115.1 (d, $^2J_{C-F}$ = 22.1 Hz, CH), 114.4 (CH), 109.5 (C), 92.74 (C), 92.68 (C), 92.6 (C), 91.1 (C), 87.96 (C), 87.94 (C), 74.8 (CH), 66.8 (CH_2), 26.7 (CH_3). **^{19}F NMR (376 MHz, $CDCl_3$)** δ -111.28. **HRMS (ESI):** *m/z* $[M+Na]^+$ calcd for $C_{53}H_{34}O_4F_2Na$: 795.2323; found: 795.2331.

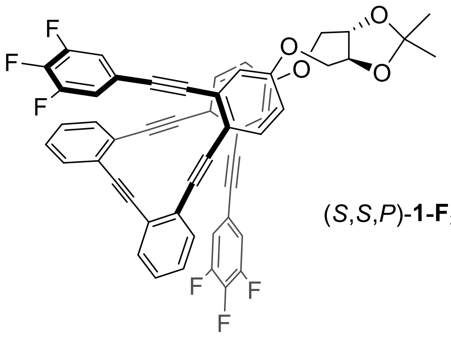

(S,S,P)-**1-F$_3$**

^{1}H NMR (400 MHz, CDCl$_3$) δ 7.47 (dd, J = 7.5, 1.5 Hz, 2H), 7.32 (dd, J = 7.4, 1.6 Hz, 2H), 7.30 – 7.27 (m, 2H), 7.21 (td, J = 8.3, 7.9, 2.0 Hz, 2H), 7.17 (td, J = 7.5, 1.6 Hz, 2H), 6.97 (dd, J = 7.8, 6.4 Hz, 4H), 6.51 – 6.49 (m, 4H), 4.34 – 4.27 (m, 4H), 4.27 – 4.21 (m, 2H), 1.45 (s, 6H). **^{13}C NMR (101 MHz, CDCl$_3$)** δ 157.2 (C), 150.4 (ddd, J = 250.1, 10.3, 4.4 Hz, C), 139.7 (dt, $J_{C\text{-}F}$ = 254.9, 15.5 Hz, C), 133.7 (CH), 132.6 (CH), 127.9 (CH), 127.7 (CH), 125.6 (C), 124.6 (d, $J_{C\text{-}F}$ = 5.6 Hz, C), 119.3 (C), 118.9 (td, $J_{C\text{-}F}$ = 10.2, 4.9 Hz, C), 117.6 (CH), 116.1 (dd, $J_{C\text{-}F}$ = 17.0, 5.5 Hz, CH), 113.9 (CH), 109.4 (C), 92.4 (C), 92.1 (C), 91.2 (C), 90.6 (q, $J_{C\text{-}F}$ = 3.2 Hz, C), 89.44 (C), 89.43 (C), 74.6 (CH), 66.6 (CH$_2$), 26.7 (CH$_3$). **^{19}F NMR (376 MHz, CDCl$_3$)** δ –134.60 (d, J = 20.7 Hz, 4F), –159.23 (t, J = 20.5 Hz, 2F). **HRMS (ESI):** *m/z* [M+Na]$^+$ calcd for $C_{53}H_{30}F_6O_4Na$: 867.1946; found: 867.1955.

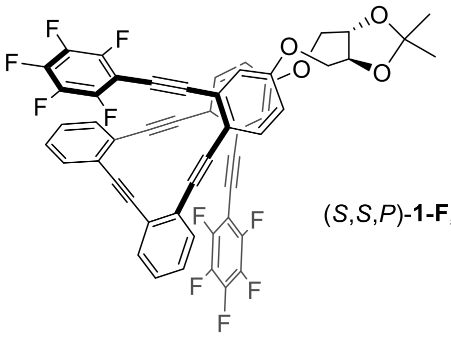

(S,S,P)-**1-F$_5$**

^{1}H NMR (400 MHz, CDCl$_3$) δ 7.50 (d, J = 8.6 Hz, 2H), 7.44 – 7.37 (m, 4H), 7.24 – 7.20 (m, 4H), 6.75 (dd, J = 8.7, 2.7 Hz, 2H), 6.50 (d, J = 2.6 Hz, 2H), 4.44 – 4.30 (m, 6H), 1.53 (s, 6H). **^{13}C NMR (101 MHz, CDCl$_3$)** δ 157.4 (C), 134.3 (CH), 133.5 (CH), 132.7 (CH), 127.8 (CH), 127.7 (CH), 125.3 (C), 124.9 (C), 124.5 (C), 119.8 (C), 118.6 (CH), 113.6 (CH), 109.7 (C), 92.01 (C), 91.95 (C), 91.2 (C), 74.9 (CH), 66.9 (CH$_2$), 27.0 (CH$_3$). Quaternary carbons attached to fluorine atoms are not listed due to their low intensity as a result of multiple signal splitting. **^{19}F NMR (376 MHz, CDCl$_3$)** δ –134.75 (dd, J = 21.5, 7.2 Hz, 4F), –153.12 (t, J = 21.0 Hz, 2F), –162.72 (td, J = 21.5, 7.2 Hz, 4F). **HRMS (ESI):** *m/z* [M+Na]$^+$ calcd for $C_{53}H_{26}O_4F_{10}Na$: 939.1569; found: 939.1567.

3. NMR SPECTRA OF NEW COMPOUNDS

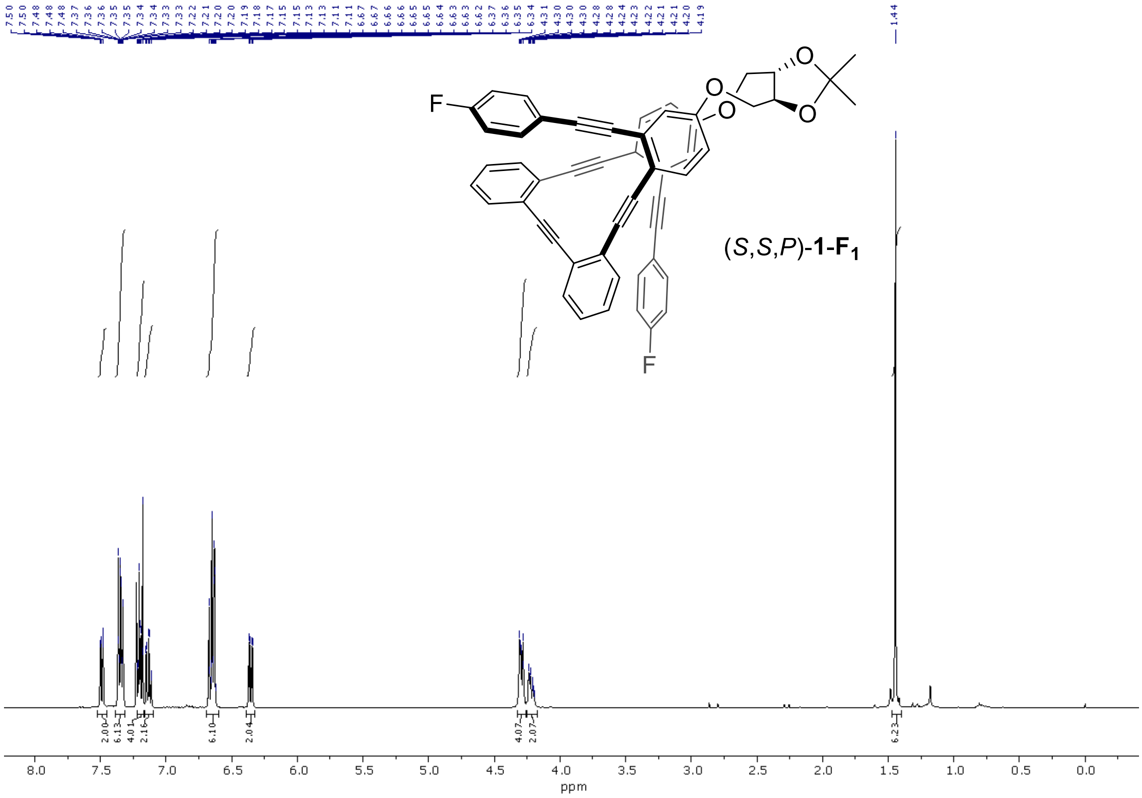

Figure S1. ^{1}H-NMR (400 MHz) spectrum of (*S*,*S*,*P*)-**1**-**F$_1$** in $CDCl_3$

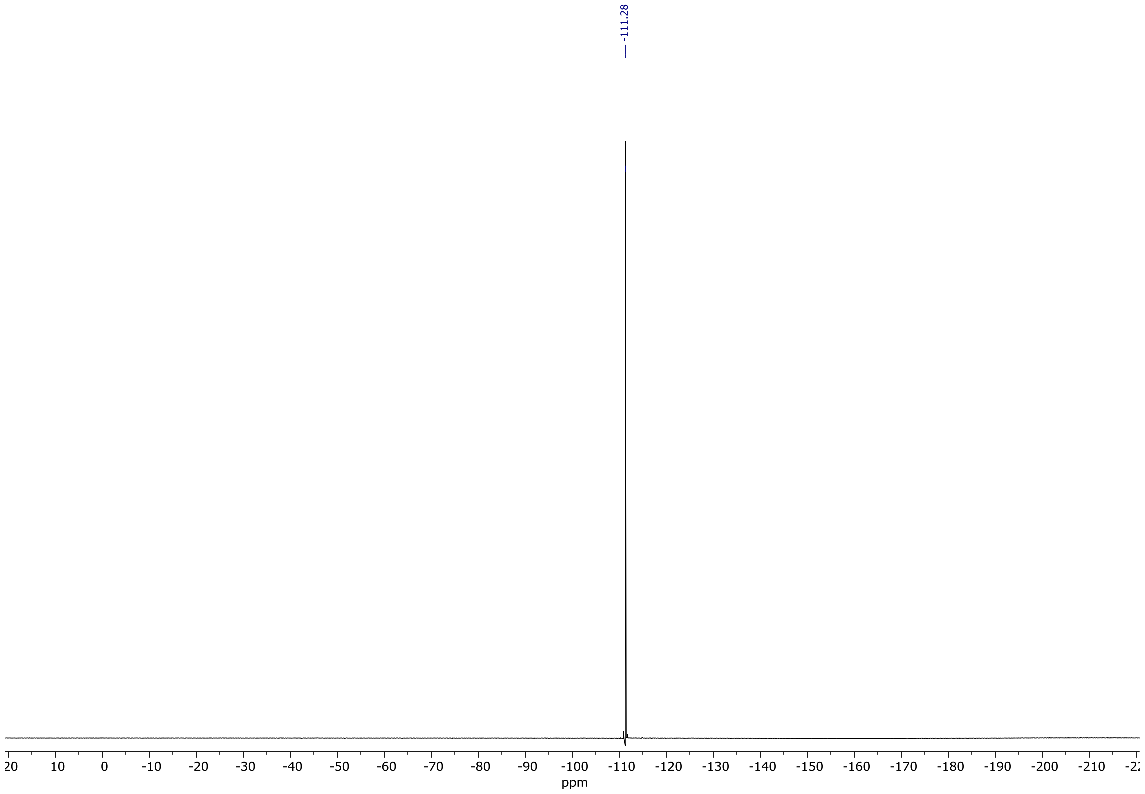

Figure S2. ^{19}F-NMR (376 MHz) spectrum of (*S*,*S*,*P*)-**1**-**F$_1$** in $CDCl_3$

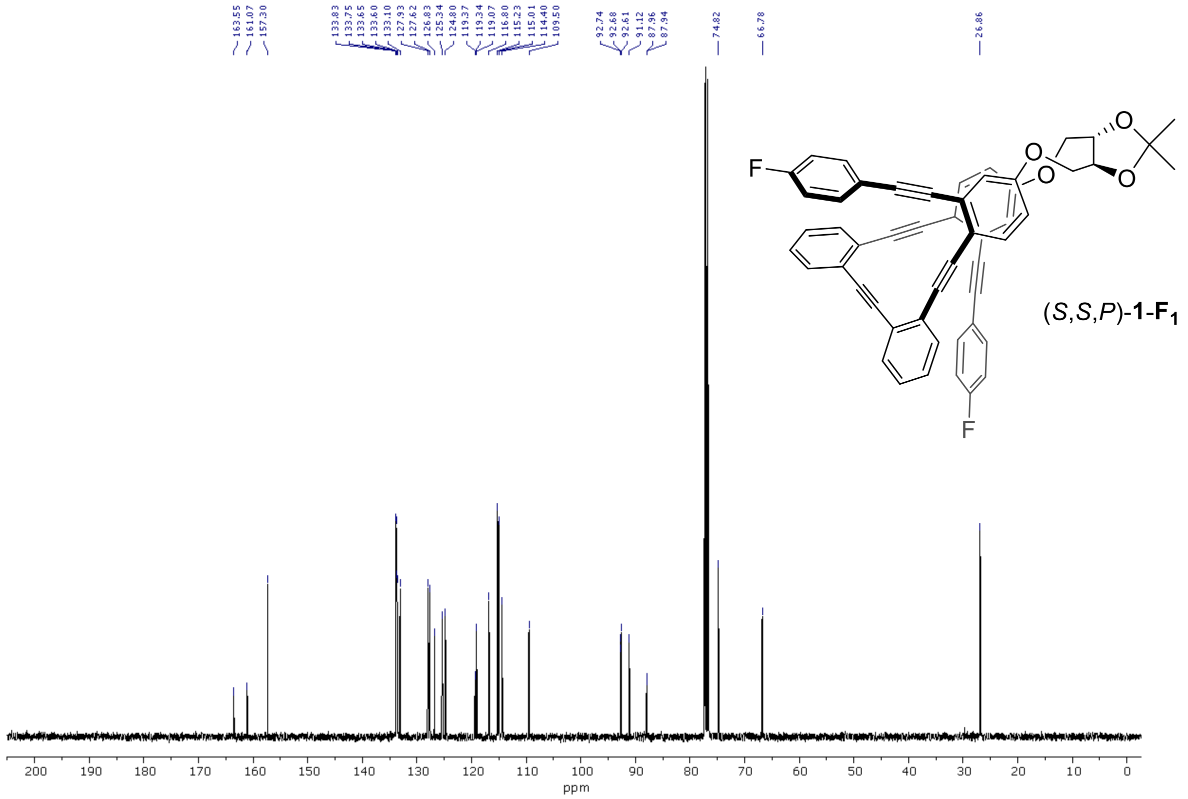

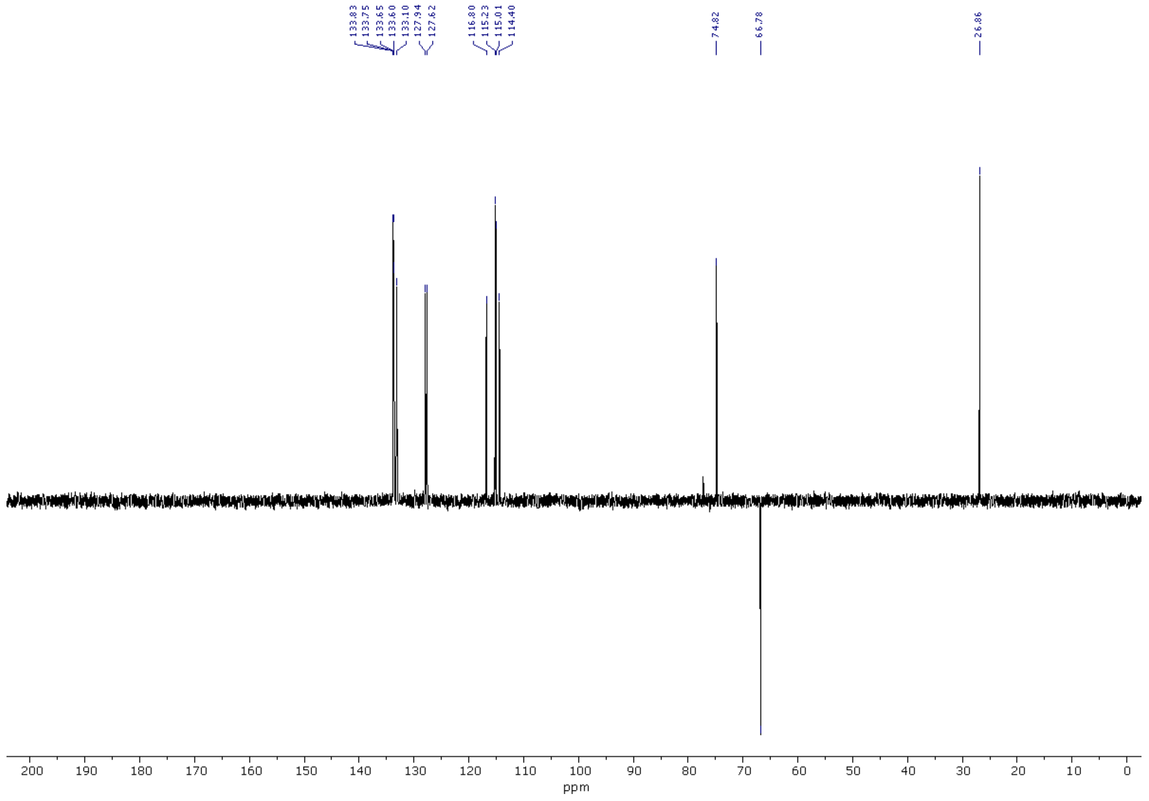

Figure S3. ^{13}C-NMR (101 MHz) and DEPT-135 spectra of (*S*,*S*,*P*)-**1**-**F$_1$** in $CDCl_3$

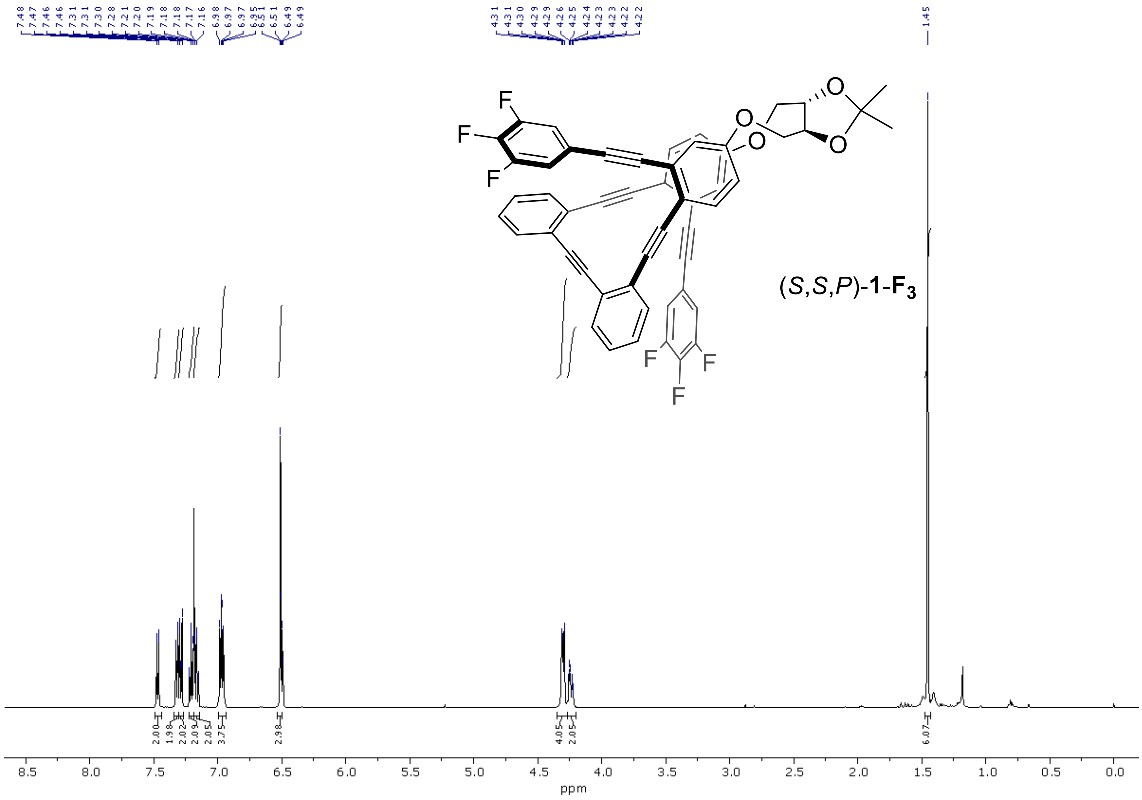

Figure S4. ^{1}H-NMR (400 MHz) spectrum of (*S*,*S*,*P*)-**1**-**F$_3$** in $CDCl_3$

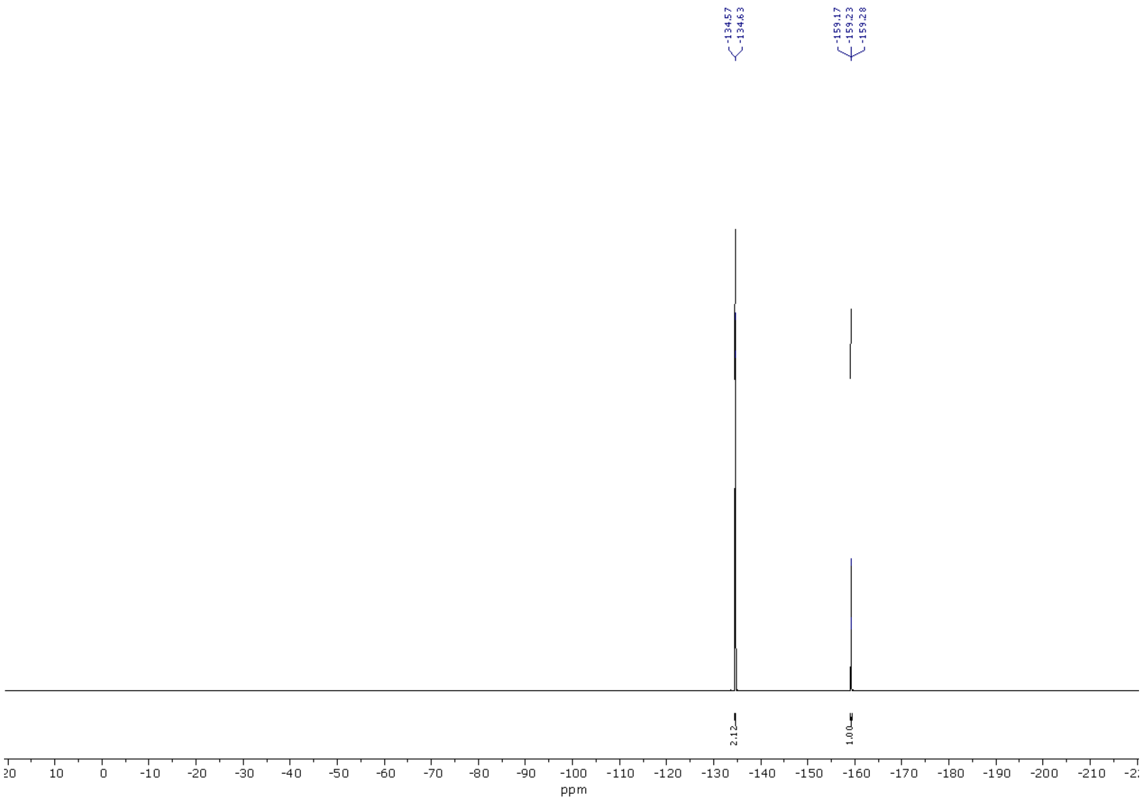

Figure S5. ^{19}F-NMR (376 MHz) spectrum of (*S*,*S*,*P*)-**1**-**F$_3$** in $CDCl_3$

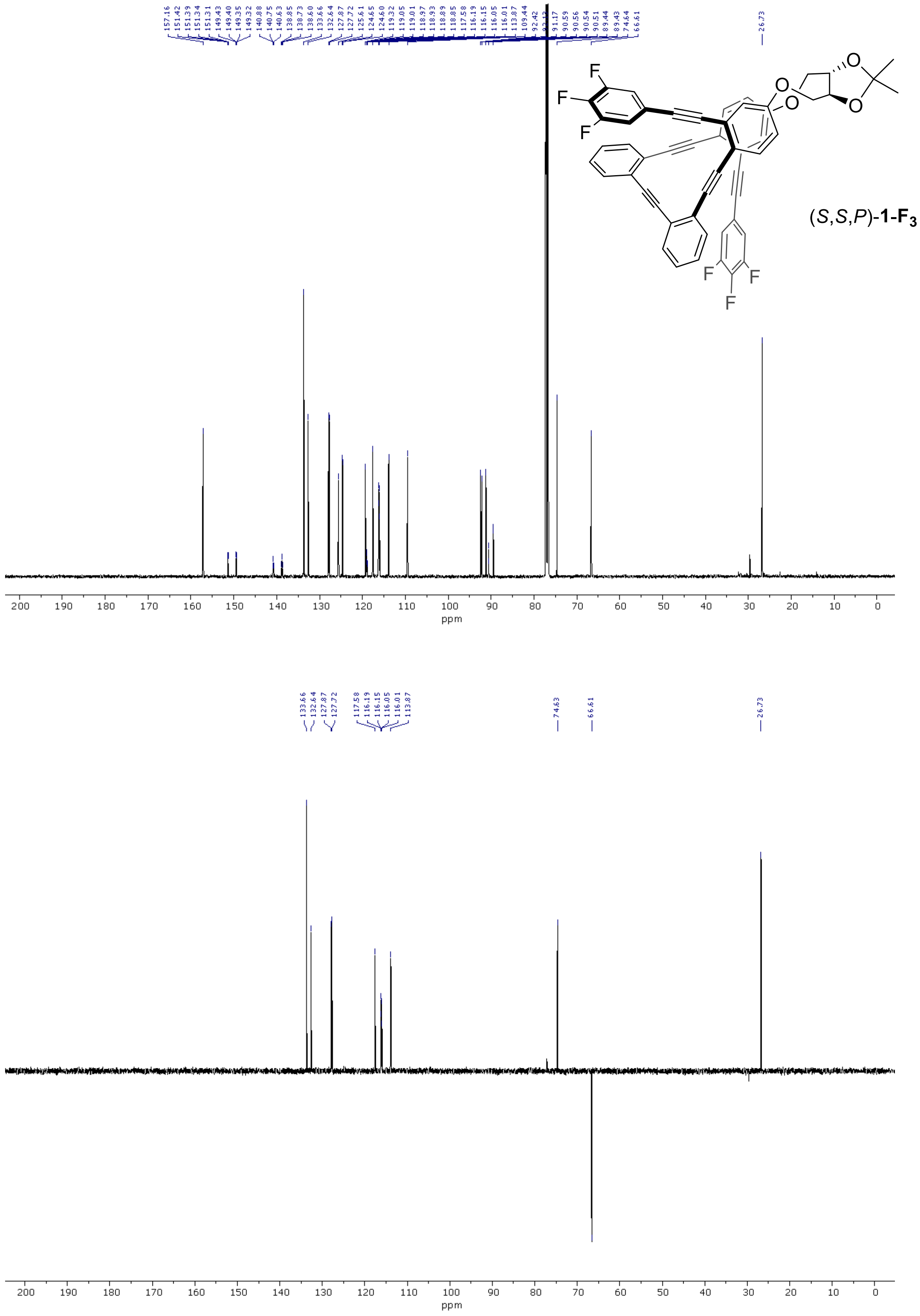

Figure S6. ^{13}C-NMR (101 MHz) and DEPT-135 spectra of (*S*,*S*,*P*)-**1**-$\mathbf{F_3}$ in $CDCl_3$

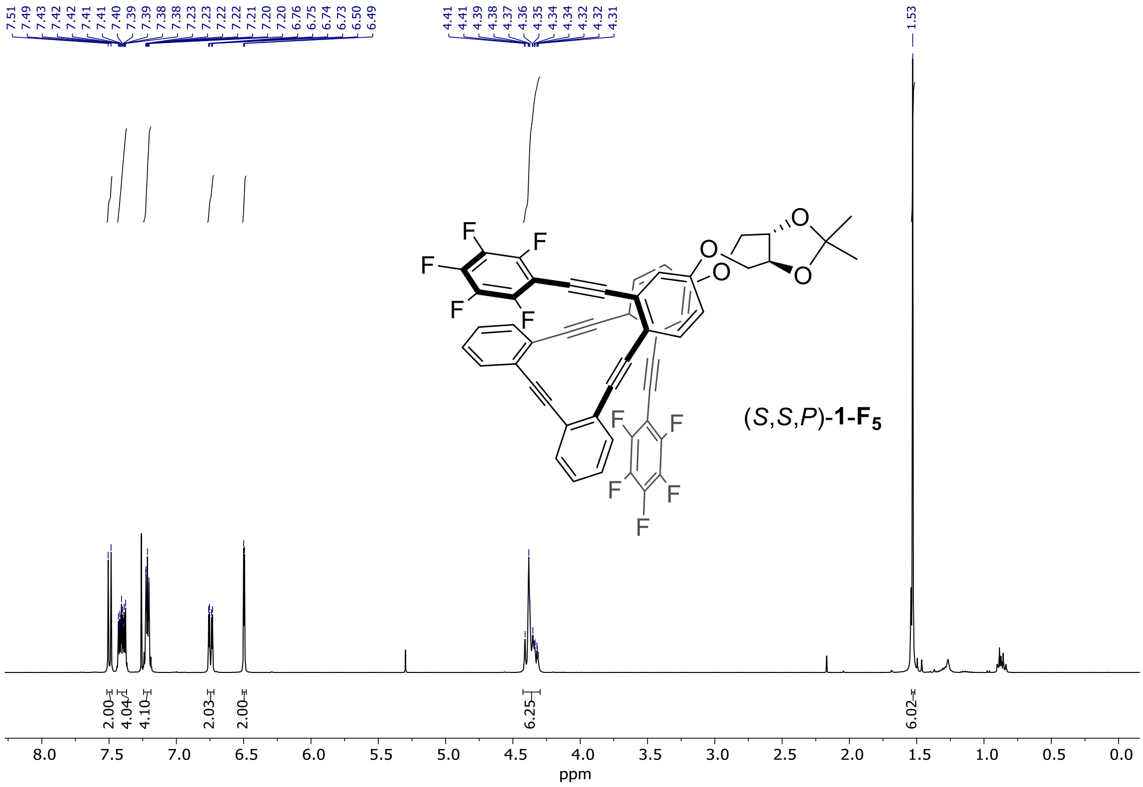

Figure S7. ^{1}H-NMR (400 MHz) spectrum of (*S*,*S*,*P*)-**1**-**F$_5$** in CDCl$_3$

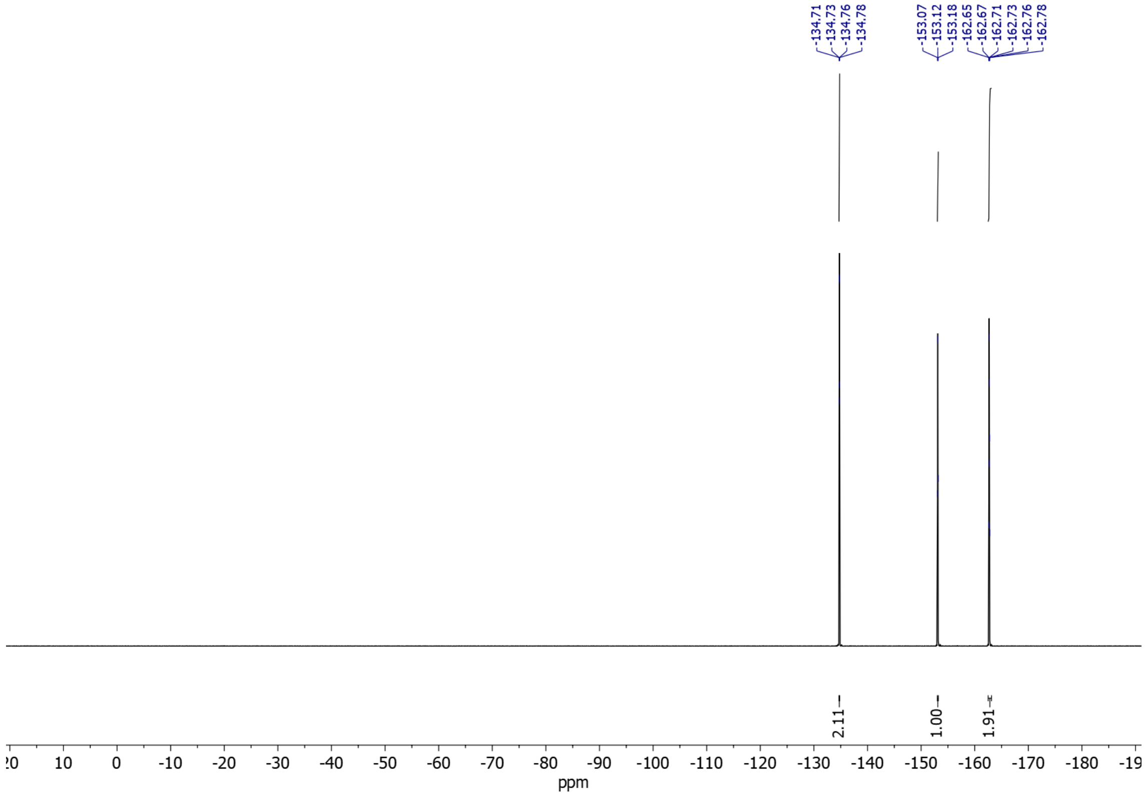

Figure S8. ^{19}F-NMR (376 MHz) spectrum of (*S*,*S*,*P*)-**1**-**F$_5$** in CDCl$_3$

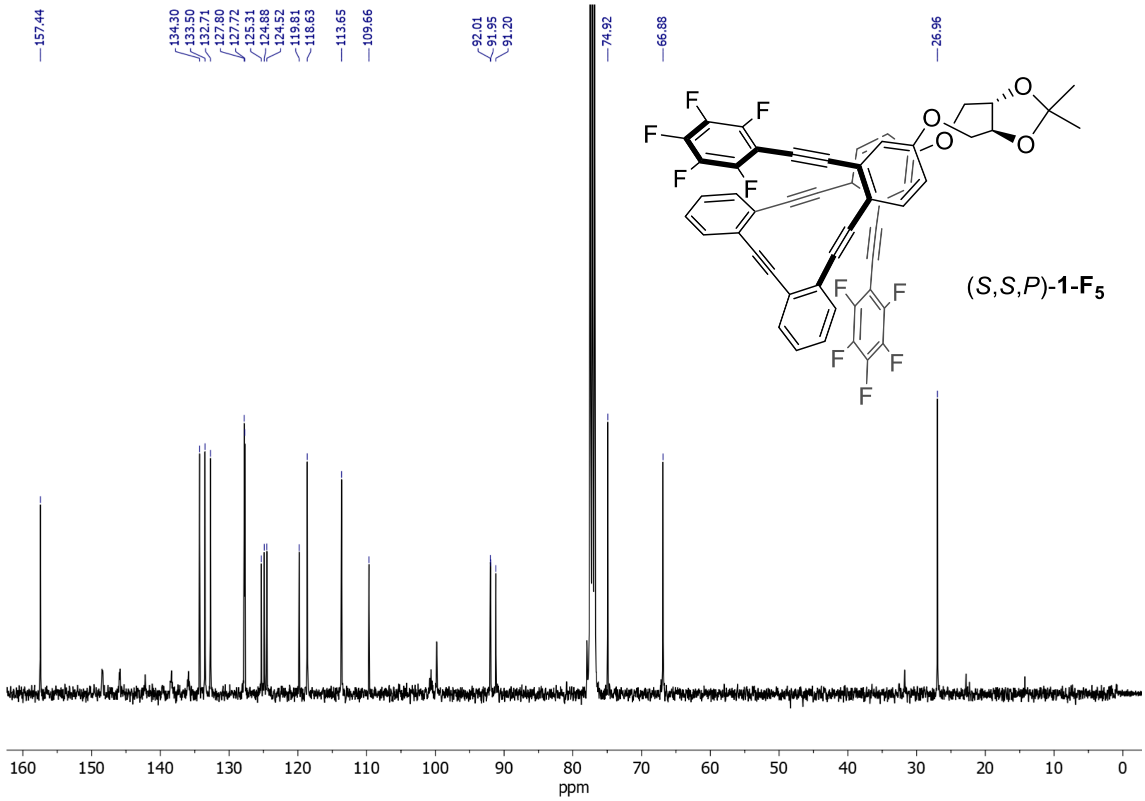

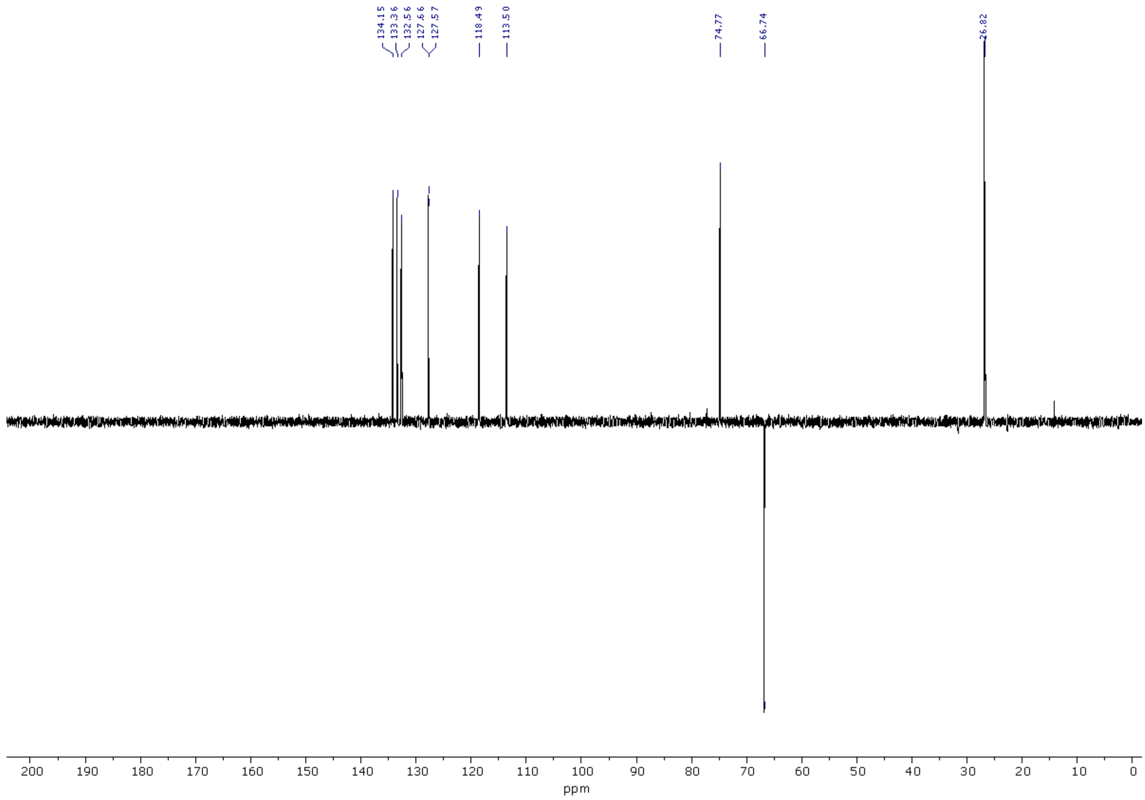

Figure S9. ^{13}C-NMR (101 MHz) and DEPT-135 spectra of (*S*,*S*,*P*)-**1**-$\mathbf{F_5}$ in $CDCl_3$

4. PHOTOPHYSICAL STUDIES

4.1. Absorbance and Emission Spectra of (*S*,*S*,*P*)-1-F_5 in Different Solvents

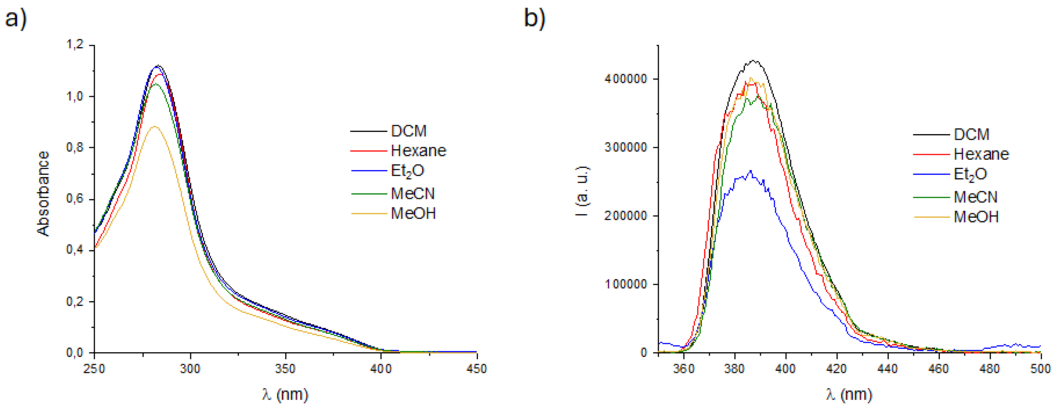

Figure S10. a) UV-Vis and b) fluorescence spectra of (*S*,*S*,*P*)-**1**-**F_5** in different solvents.

4.2. Lifetimes and Quantum Yields of (*S*,*S*,*P*)-1-F_n

Time-resolved fluorescence decay traces were collected via the time-correlated single photon counting (TCSPC) method using a FluoTime 200 fluorometer (PicoQuant GmbH). The excitation source was a 320 nm LED (for (*S*,*S*,*P*)-**1-F_1** and (*S*,*S*,*P*)-**1-F_3**) and a 375 nm laser (for (*S*,*S*,*P*)-**1-F_5**) using a 10 MHz excitation frequency. The full width at half maximum (fwhm) of the laser pulses was around 40 ps. The fluorescence emission was collected at a 90° geometry, focused on the detector after crossing through a polarizer (set at the magic angle), 2 mm slits, and a 2 nm bandwidth monochromator. TCSPC was achieved by a TimeHarp200 board, set at 36 ps/channel. Fluorescence decay traces were collected for the necessary time to reach 20000 counts at the peak channel. For the analyzed compounds, decay traces were collected at three different wavelengths within ± 5nm (Δλ = - 5, 0, +5 nm) of the maximum of the main emission peak.

The fluorescence decay traces were fitted to a bi-exponential function by using an iterative reconvolution method based on the Levenberg-Marquardt algorithm for nonlinear least-squares error minimization deconvolution (FluoFit 4.4 package, PicoQuant GmbH). For each sample, the decay traces were fitted globally with the decay times linked as shared parameters, whereas the pre-exponential factors were local adjustable parameters. The quality of fittings was assessed by the value of the reduced chi-squared, χ^2, parameter and random distributions of the weighted residuals and the autocorrelation functions.

Quantum yields were determined by measuring both absorbance and fluorescence of compounds (*S*,*S*,*P*)-**1**-**F**$_n$ in CH_2Cl_2 and MeOH, and (*S*,*S*,*P*)-**1**-**F**$_5$ also in hexane and MeCN, using quinine sulfate in 0.1 M H_2SO_4 as standard (Φ_r = 0.54).[S4] For the relative determination of the fluorescence quantum yield Φ in a series of solvents, eq. 1 was used.[S5]

$$\Phi_x = \Phi_r \times \frac{F_x}{F_r} \times \frac{1 - 10^{-A_r(\lambda_{ex})}}{1 - 10^{-A_x(\lambda_{ex})}} \times \frac{n_x^2}{n_r^2} \quad \text{(Eq. 1)}$$

The subscripts *x* and *r* refer respectively to the sample and a reference (standard) fluorophore with known quantum yield Φ_r in a specific solvent; *F* stands for the spectrally corrected, integrated fluorescence spectra; $A(\lambda_{ex})$ denotes the absorbance at the used excitation wavelength λ_{ex}; and *n* represents the refractive index of the solvent (in principle at the average emission wavelength). To minimize inner filter effects, the absorbance at the excitation wavelength λ_{ex} was kept under 0.1. The measurements were performed using 10×10 mm cuvettes.

Table S1 summarizes the fluorescence lifetimes and quantum yields obtained for described compounds.

Table S1. Fluorescence quantum yields (QY) and lifetimes (τ) of compounds (*S*,*S*,*P*)-**1**-**F**$_n$ in different solvents.

SOLVENT	COMPOUND	QY / %	τ_1 / ns	τ_2 / ns	τ_{av} / ns
	(*S*,*S*,*P*)-**1**-**F**$_1$	29.7±0.5	4.16±0.02	2.49±0.06	4.22
CH_2Cl_2	(*S*,*S*,*P*)-**1**-**F**$_3$	20.0±0.3	4.51±0.03	2.13±0.03	3.77
	(*S*,*S*,*P*)-**1**-**F**$_5$	21.4±2.3	4.68±0.02	1.79±0.19	4.54
	(*S*,*S*,*P*)-**1**-**F**$_1$	25.7±0.4	4.72±0.02	2.67±0.07	4.38
MeOH	(*S*,*S*,*P*)-**1**-**F**$_3$	22.0±0.3	4.69±0.02	2.38±0.10	4.43
	(*S*,*S*,*P*)-**1**-**F**$_5$	16.1±0.6	4.68±0.02	1.38±0.21	4.59
Hexane	(*S*,*S*,*P*)-**1**-**F**$_5$	18.5 ± 1.0	4.04±0.02	1.56±0.17	3.95
MeCN	(*S*,*S*,*P*)-**1**-**F**$_5$	15.6 ± 0.8	4.70±0.02	1.25±0.18	4.61

4.3. CD and CPL Spectra

4.3.1. Comparison of CD and CPL spectra of (*S*,*S*,*P*)-**1** and (*S*,*S*,*P*)-**1-Fn**

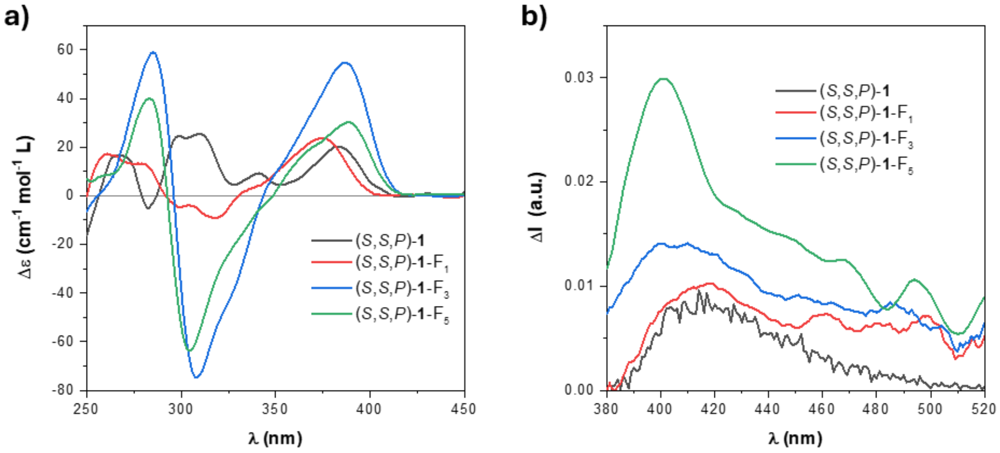

Figure S11. a) ECD and b) CPL spectra of all described compounds in CH_2Cl_2.

4.3.2. CD Spectra of (*S*,*S*,*P*)-**1** in Different Solvents

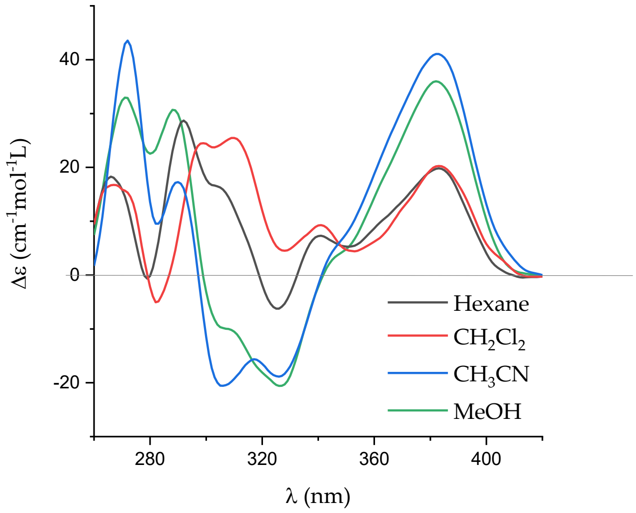

Figure S12. a) ECD spectra of compound (*S*,*S*,*P*)-**1** in different solvents.

4.3.3. CD and CPL Spectra of Both Enantiomers of (*S*,*S*,*P*)-**1-F$_5$** in Different Solvents

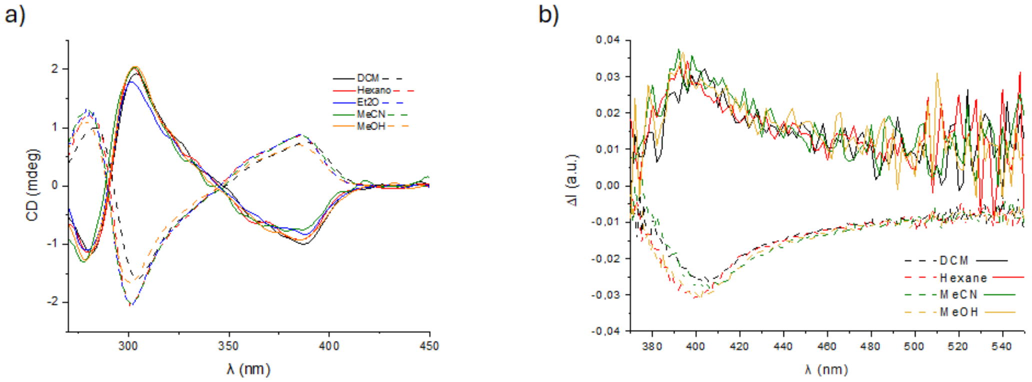

Figure S13. a) ECD of (*S*,*S*,*P*)-**1**-**F$_5$** (solid lines) and (*R*,*R*,*M*)-**1**-**F$_5$** (dashed lines) in different solvents. b) CPL of (*S*,*S*,*P*)-**1**-**F$_5$** (solid lines) and (*R*,*R*,*M*)-**1**-**F$_5$** (dashed lines) in different solvents.

4.4. Photophysical measurements for 1-F$_1$ and 1-F$_3$ in MeOH

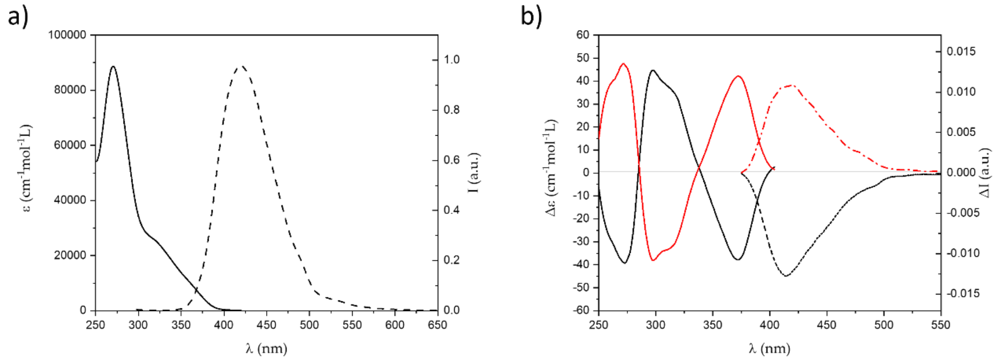

Figure S14. a) UV-Vis (solid line) and fluorescence (dashed line) spectra of (*S*,*S*,*P*)-**1**-**F$_1$**. b) ECD (solid line) and CPL (dashed line) of (*S*,*S*,*P*)-**1**-**F$_1$** (red line) and (*R*,*R*,*M*)-**1**-**F$_1$** (black line). All the spectra were recorded using 1.5x10^{-5}M solutions in CH_2Cl_2.

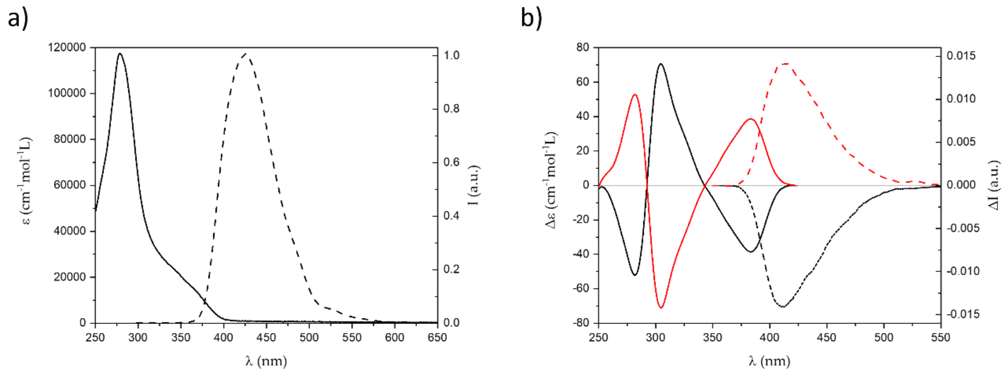

Figure S15. a) UV-Vis (solid line) and fluorescence (dashed line) spectra of (*S*,*S*,*P*)-**1**-**F$_3$**. b) ECD (solid line) and CPL (dashed line) of (*S*,*S*,*P*)-**1**-**F$_3$** (red line) and (*R*,*R*,*M*)-**1**-**F$_3$** (black line). All the spectra were recorded using 1.5x10^{-5}M solutions in CH_2Cl_2.

4.5. Luminescence Dissymmetry Factor (g_{lum}) Graphics

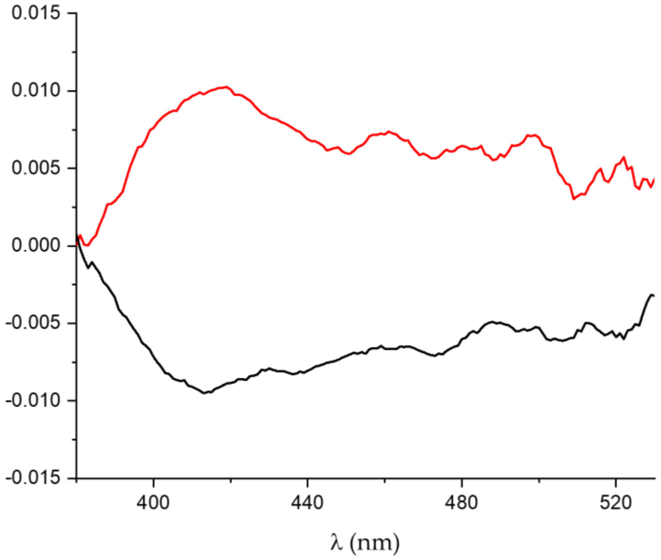

Figure S16. g_{lum} values for (*S*,*S*,*P*)-**1**-**F**$_1$ (red line) and (*R*,*R*,*M*)-**1**-**F**$_1$ (black line) in DCM

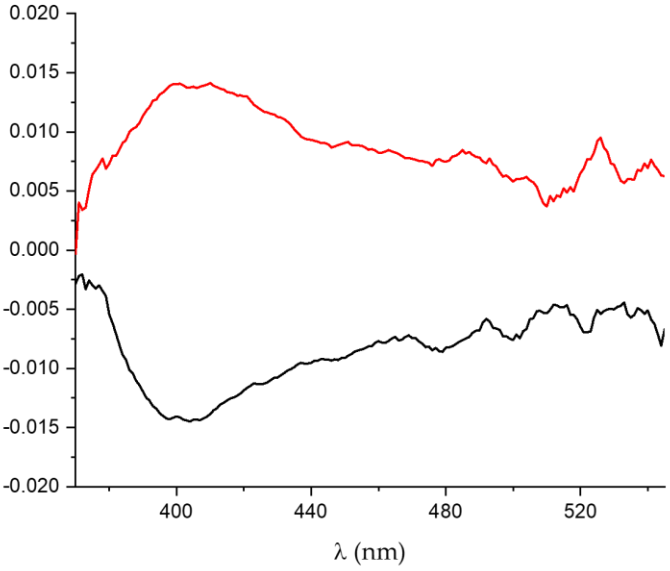

Figure S17. g_{lum} values for (*S*,*S*,*P*)-**1**-**F**$_3$ (red line) and (*R*,*R*,*M*)-**1**-**F**$_3$ (black line) in DCM

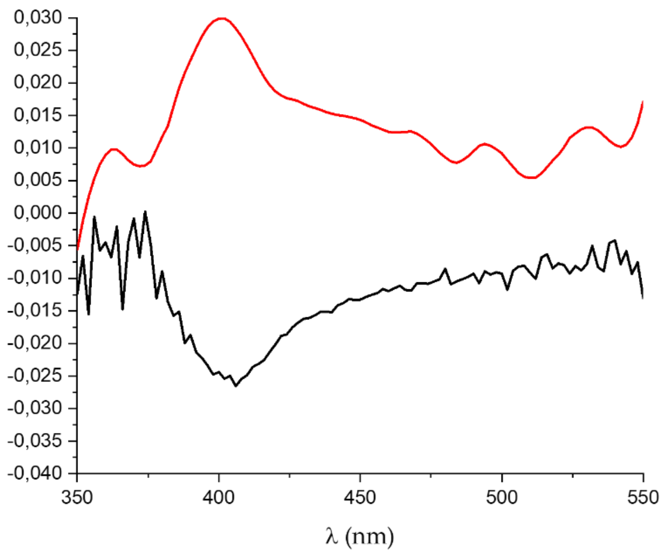

Figure S18. g_{lum} values for (*S*,*S*,*P*)-**1**-**F$_5$** (red line) and (*R*,*R*,*M*)-**1**-**F$_5$** (black line) in DCM

4.6. ECD Titration of (*S*,*S*,*P*)-1 and (*S*,*S*,*P*)-1-F$_n$ with Ag(I)

Titrations of compounds (*S*,*S*,*P*)-**1,** (*S*,*S*,*P*)-**1**-**F$_1$**, (*S*,*S*,*P*)-**1**-**F$_3$** and (*S*,*S*,*P*)-**1**-**F$_5$** were carried out by progressive addition of constant quantities of $AgBF_4$ (2.5×10^{-3} M) to a solution of (*S*,*S*,*P*)-**1**, (*S*,*S*,*P*)-**1**-**F$_n$** in a 9:1 mixture of CH_2Cl_2:acetone (2.5×10^{-5}M).

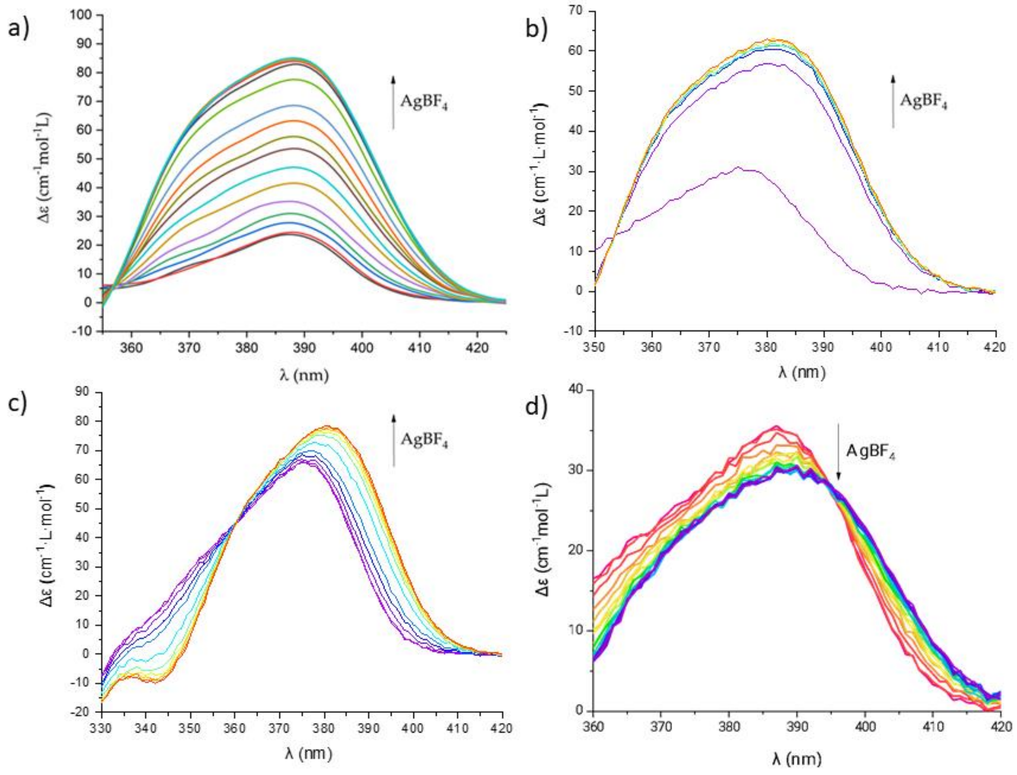

Figure S19. ECD titration of compounds a) (*S,S,P*)-**1**, b) (*S,S,P*)-**1**-**F$_1$**, c) (*S,S,P*)-**1**-**F$_3$** and d) (*S,S,P*)-**1**-**F$_5$** with $AgBF_4$.

5. THEORETICAL CALCULATIONS

5.1. Conformers' optimization

For both compounds (*S*,*S*,*P*)-**1** and (*S*,*S*,*P*)-**1**-**F_5**, preliminary conformational search was carried out using the CREST protocol, based on the semiempirical extended Tight-Binding approach GFN2-xTB.[S6] The structures thus obtained have been optimized at PBE/cc-pVDZ level with empirical dispersion corrections D3BJ,[S7] adopting iefpcm for the solvent (CH_2Cl_2) as implemented within Gaussian16 package, [S8] also vibrational frequency. analysis has been performed. For all optimized structures no imaginary frequency has been found so they can be assumed as true minima.Examining the $\phi1$, $\phi2$, $\phi3$, $\phi4$ and $\phi5$ dihedral angles of the aliphatic staple and the *o*-OPE backbone described by $\tau1$, $\tau2$ and $\tau3$ (see Figure S18), one may appreciate that the geometries are very similar in the two cases, only the energy order is quite different: the lowest energy structure is practically the same for (*S*,*S*,*P*)-**1** and (*S*,*S*,*P*)-**1**-**F_5** but the first "unfolded structure "f" for (*S*,*S*,*P*)-**1**-**F_5** is at higher energy (with other folded conformers differing only for the staple conformation) then the same type of "f" conformer which is the second one at 0.74 kcal/mol for (*S*,*S*,*P*)-**1**.

These parameters together with energy values and Boltzmann populations are shown in Table S3 for (*S*,*S*,*P*)-**1** and Table S2 for (*S*,*S*,*P*)-**1**-**F_5**.

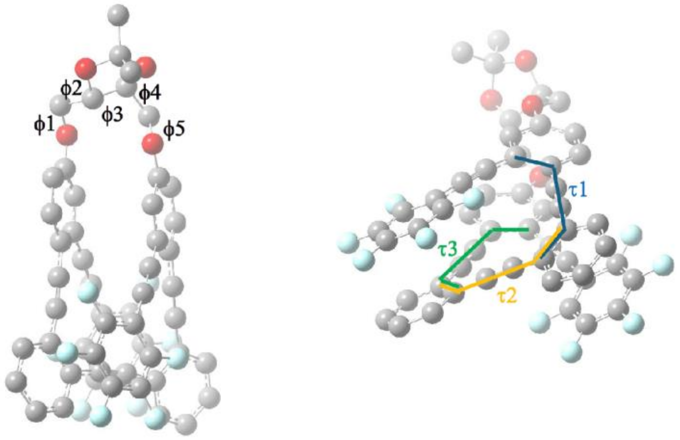

Figure S20. Representation of the main parameters evaluated in the conformational study of *o*-OPEs.

Table S*2*. Energy values, Boltzmann populations, dihedral angles. PBE/cc-pvdz empiricaldispersion corrections, iefpcm dichloromethane, (*S*,*S*,*P*)-**1**-**F$_5$** compound (τi acute angles correspond to folded structures, evidenced in light blue in the table)

	E	pop	G	pop	φ1	φ2	φ3	φ4	φ5	τ1	τ2	τ3
a	0.00	*95.8%*	0.00	*94.3%*	154	-45	-84	65	78	30	21	36
b	2.05	*3.0%*	1.81	*4.4%*	80	50	-148	50	80	30	16	30
c	2.85	*0.8%*	3.08	*0.5%*	79	73	-107	82	-127	39	22	34
d	3.37	*0.3%*	2.89	*0.7%*	-152	107	-92	46	80	30	21	38
e	4.49	*0.0%*	4.69	*0.0%*	-65	-177	-98	66	-113	36	22	31
f	5.12	*0.0%*	4.91	*0.0%*	71	62	-87	-45	161	-145	23	30
g	6.65	*0.0%*	6.66	*0.0%*	-99	69	-90	148	-150	150	-29	-23
h	6.89	*0.0%*	6.89	*0.0%*	-54	-177	-101	67	-98	19	33	-162
i	6.96	*0.0%*	5.52	*0.0%*	-147	88	-77	91	-159	152	-24	-29
j	7.63	*0.0%*	7.27	*0.0%*	-151	148	-89	75	-97	152	-25	-35
k	8.66	*0.0%*	7.43	*0.0%*	113	-52	-103	51	89	149	-20	-34
l	8.83	*0.0%*	9.13	*0.0%*	-72	170	-94	-48	137	33	20	-161
m	14.17	*0.0%*	10.11	*0.0%*	68	67	-148	65	-90	163	-28	162
n	17.30	*0.0%*	12.10	*0.0%*	113	-53	-106	47	78	153	-21	163

Table S*3*. Energy values, Boltzmann populations, dihedral angles. PBE/cc-pvdz empiricaldispersion corrections, iefpcm dichloromethane, (*S*,*S*,*P*)-**1** compound (τi acute angles correspond to folded structures, evidenced in light blue in the table)

	E	pop	G	pop	φ1	φ2	φ3	φ4	φ5	τ1	τ2	τ3
a	0.00	*73.6%*	0.00	*74.2%*	157	-46	-85	65	75	27	26	30
f	0.74	*21.0%*	0.70	*22.8%*	75	64	-84	-45	156	-147	24	24
b	1.65	*4.5%*	2.27	*1.6%*	79	51	-148	51	79	30	27	30
c	3.39	*0.2%*	2.89	*0.6%*	79	75	-108	81	-123	33	20	30
d	3.61	*0.2%*	3.14	*0.4%*	-157	113	-91	43	77	25	27	32
i	3.81	*0.1%*	4.95	*0.0%*	-150	88	-76	90	-158	149	-22	-28
k	3.86	*0.1%*	3.61	*0.2%*	120	-52	-102	48	84	147	-18	-37
g	3.98	*0.1%*	3.94	*0.1%*	-99	70	-90	149	-149	146	-27	-23
e	4.53	*0.0%*	3.91	*0.1%*	-65	-176	-98	67	-111	29	21	26
j	4.67	*0.0%*	4.73	*0.0%*	-151	150	-89	74	-95	153	-31	-34
h	4.80	*0.0%*	4.89	*0.0%*	-57	-176	-99	66	-100	22	26	-159
l	6.77	*0.0%*	5.27	*0.0%*	-72	178	-95	-50	127	28	26	171
m	7.42	*0.0%*	5.20	*0.0%*	70	64	-148	72	-93	158	-26	156
n	9.44	*0.0%*	6.64	*0.0%*	115	-53	-104	48	80	151	-21	156

Table S*4*. Energy and free energy values, of the lowest energy structure, conformers "a" (folded), and of the first partially unfolded structure: conformer"f" for the four compounds with different degree of fluorination.

		E	G
(*S*,*S*,*P*)-**1**	conf.a	0.00	0.00
	conf.f	0.74	0.70
(*S*,*S*,*P*)-**1-F1**	conf.a	0.00	0.00
	conf.f	1.50	0.49
(*S*,*S*,*P*)-**1-F3**	conf.a	0.00	0.00
	conf.f	2.66	1.43
(*S*,*S*,*P*)-**1-F5**	conf.a	0.00	0.00
	conf.f	5.12	4.91

Table S5. Arene for (*S*,*S*,*P*)-**1**, -arene distances and arene-fluoroarene distances for the optimized lowest energy structure of (*S*,*S*,*P*)-**1-F$_1$**, (*S*,*S*,*P*)-**1-F$_3$**, and (*S*,*S*,*P*)-**1-F$_5$**.

	dist.1	dist.2
(*S*,*S*,*P*)-**1**	3.742	3.697
(*S*,*S*,*P*)-**1-F$_1$**	3.630	3.595
(*S*,*S*,*P*)-**1-F$_3$**	3.505	3.480
(*S*,*S*,*P*)-**1-F$_5$**	3.516	3.483

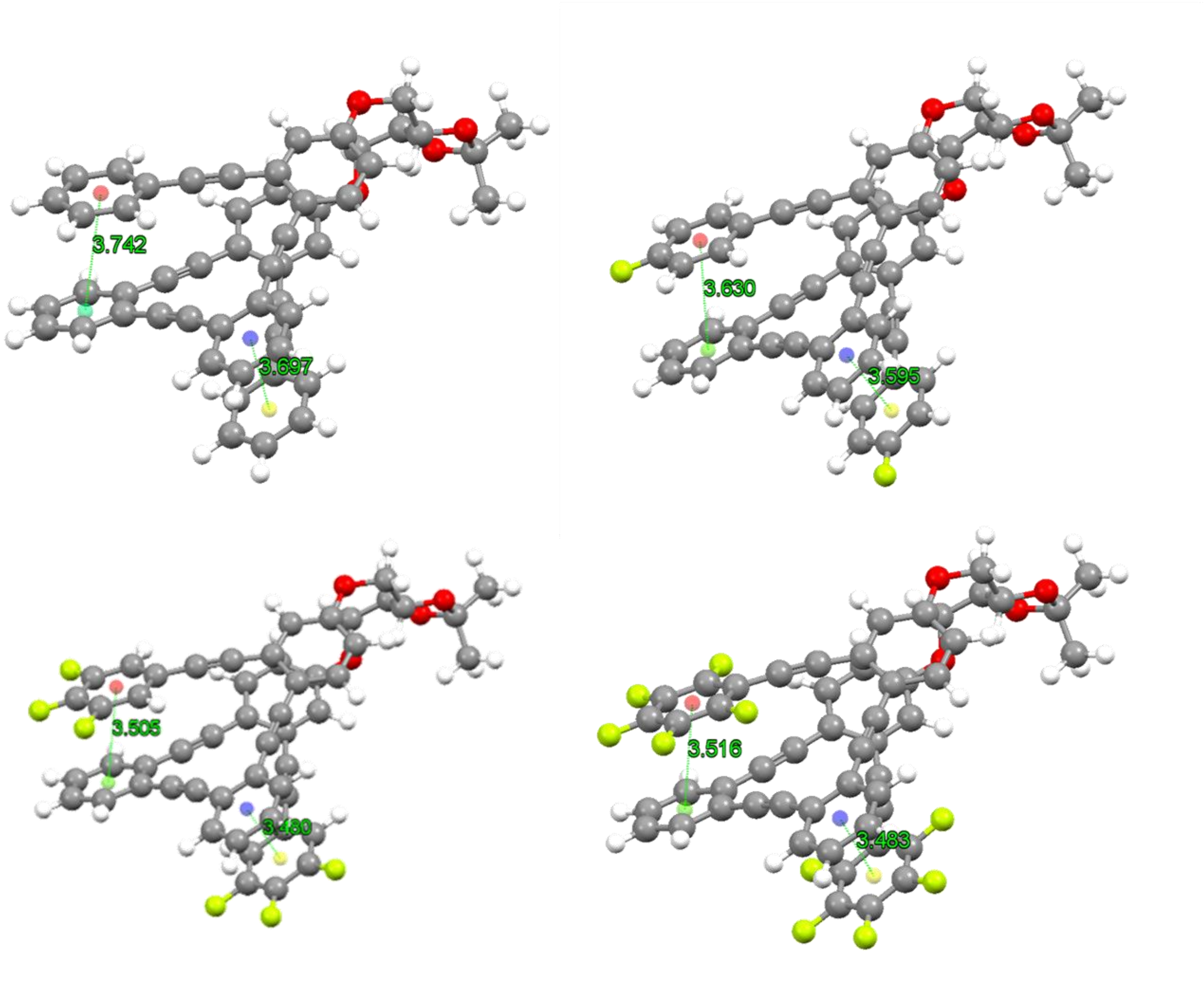

Figure S21. Lowest energy structure for *(S,S,P)*-**1**, *(S,S,P)*-**1-F$_1$**, *(S,S,P)*-**1-F$_3$**, *and (S,S,P)*-**1-F$_5$**.

5.2. Calculated spectra

Starting from the optimized structures, CD spectra have been calculated at M06-2X/cc-pVDZ level, Gaussian bands have been associated to each calculated transitions with 0.2 eV bandwidth. No wavelength shift has been applied.

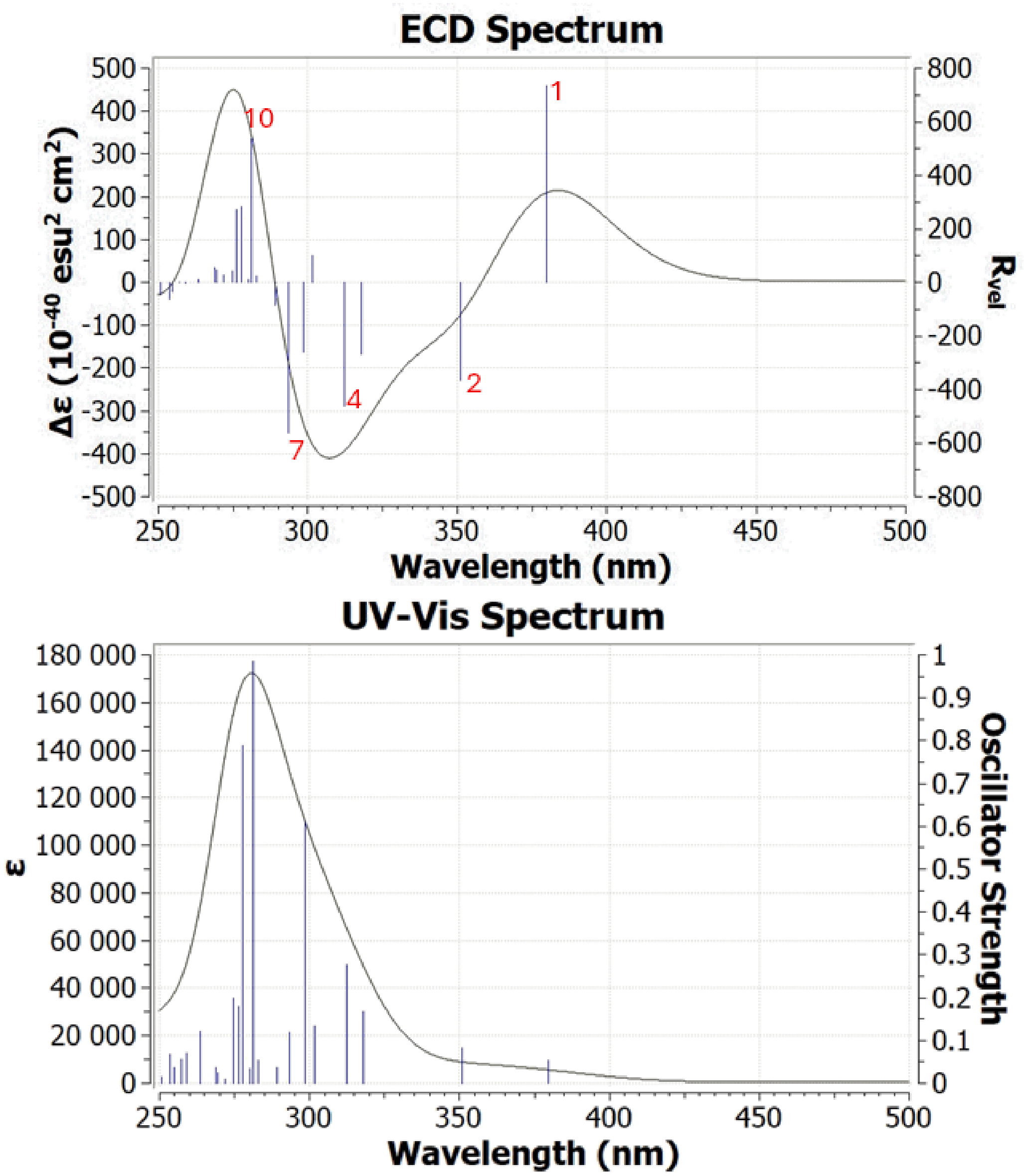

Figure S22. Calculated CD and absorption spectra of (*S*,*S*,*P*)-**1**-**F₅**, most intense CD bands are numbered, see also Table S6

Table S6. Parameters characterizing the spectroscopic properties of (*S*,*S*,*P*)-**1-F₅**: transition energy (eV) and transition wavelength (nm), Dipole Strength (D 10^{-40} esu^2 cm^2), Rotational Strengths (R 10^{-40} esu^2 cm^2), and assignment.

	eV	nm	D	R																
1	3.26	380	4.3E+04	734	233	->	234	0.66	232	->	235	0.12								-
2	3.53	351	6.2E+04	-367	233	->	235	0.62	232	->	234	0.22								-
3	3.90	318	1.1E+05	-268	232	->	234	0.59	233	->	235	-0.23	233	->	237	0.13				-
4	3.97	312	1.8E+05	-463	233	->	236	0.65	232	->	235	0.14								-
5	4.11	302	8.6E+04	102	232	->	235	0.60	233	->	236	-0.17	231	->	234	0.14				-
6	4.15	299	3.9E+05	-259	231	->	234	0.57	232	->	235	-0.17	231	->	235	-0.16				-
7	4.22	294	7.5E+04	-562	231	->	235	0.35	230	->	235	0.32	232	->	236	0.27	229	->	234	-0.22
8	4.29	289	2.2E+04	-86	230	->	234	0.36	233	->	237	0.30	232	->	236	-0.24				-
9	4.38	283	3.3E+04	23	227	->	234	0.27	233	->	237	0.22	226	->	234	-0.20				-
10	4.41	281	5.9E+05	537	232	->	236	0.46	231	->	235	-0.25	229	->	235	-0.23	230	->	234	0.18

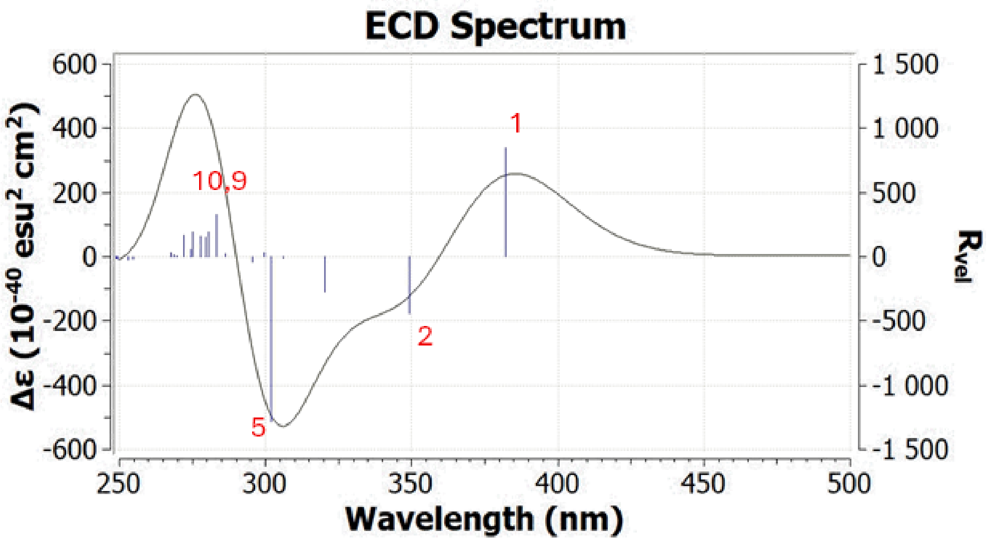

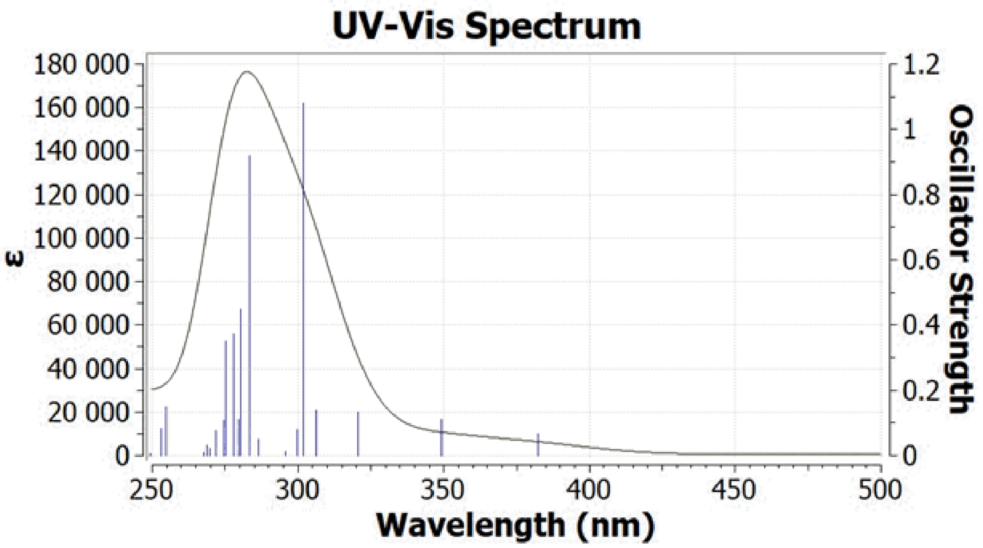

Figure S23. Calculated CD and absorption spectra of (*S*,*S*,*P*)-**1**, most intense CD bands are numbered, see also Table S7

Table S7. Parameters characterizing the spectroscopic properties of (*S*,*S*,*P*)-**1**: transition energy (eV) and transition wavelength (nm), Dipole Strength (D 10^{-40} esu^2 cm^2), Rotational Strengths (R 10^{-40} esu^2 cm^2), and assignment.

	eV	nm	D	R																
1	3.24	382	5.4E+04	853	193	->	194	0.66	192	->	195	0.14								-
2	3.55	349	8.1E+04	-444	193	->	195	0.59	192	->	194	0.30								-
3	3.87	320	9.1E+04	-272	192	->	194	0.57	193	->	195	-0.30								-
4	4.05	306	9.1E+04	-12	192	->	195	0.39	191	->	194	-0.38	193	->	196	0.28				-
5	4.11	302	6.9E+05	-1280	191	->	194	0.44	193	->	196	0.31	192	->	195	0.24	191	->	195	-0.20
6	4.14	300	5.0E+04	32	193	->	196	0.43	192	->	195	-0.35	190	->	194	0.26				
7	4.19	296	7.0E+03	-41	191	->	195	0.42	190	->	194	0.24	192	->	196	-0.19				
8	4.33	286	3.1E+04	24	190	->	194	0.37	193	->	197	-0.25	192	->	195	0.22	193	->	196	-0.22
9	4.37	283	5.5E+05	330	193	->	197	0.35	190	->	194	0.18	187	->	194	-0.18	191	->	195	-0.17
10	4.42	280	2.7E+05	191	192	->	196	0.25	182	->	194	-0.23	193	->	197	-0.20	187	->	194	-0.17

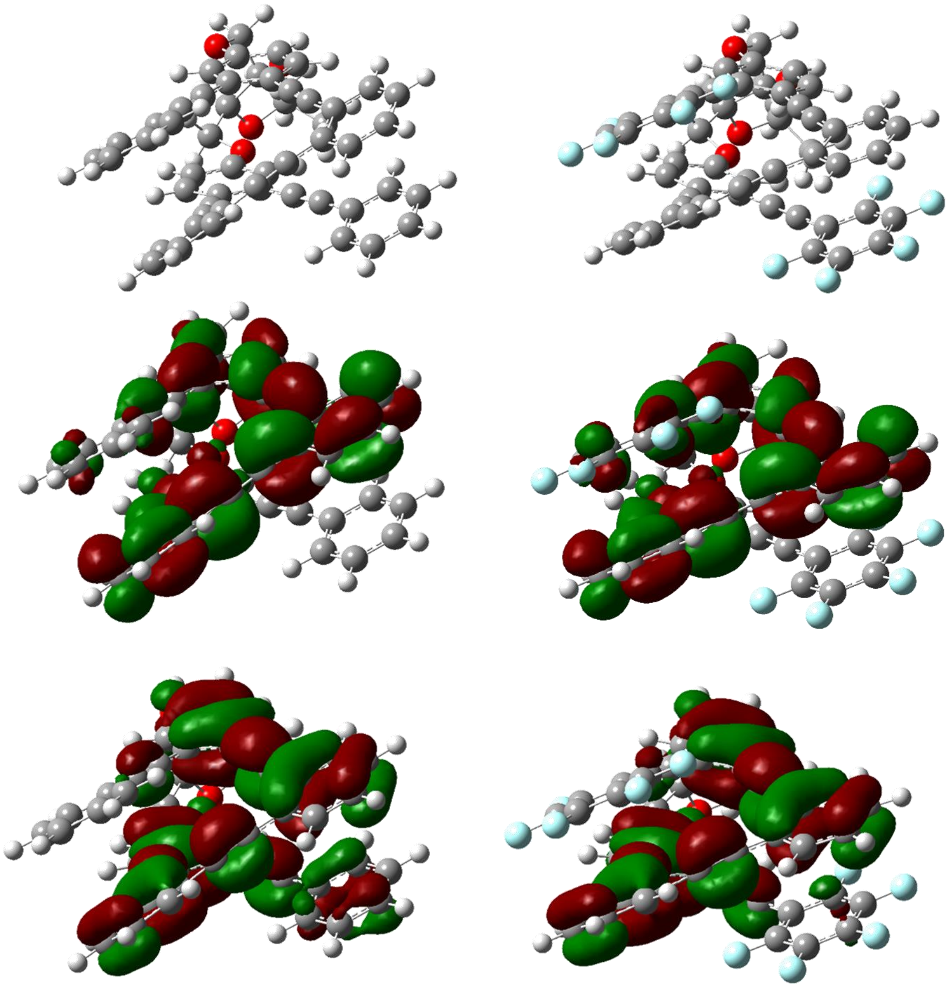

Figure S24. Contour plots of the frontier orbitals, HOMO (bottom) and LUMO (middle), of (*S*,*S*,*P*)-**1** on the left, and (*S*,*S*,*P*)-**1**-**F**$_5$, on the right.

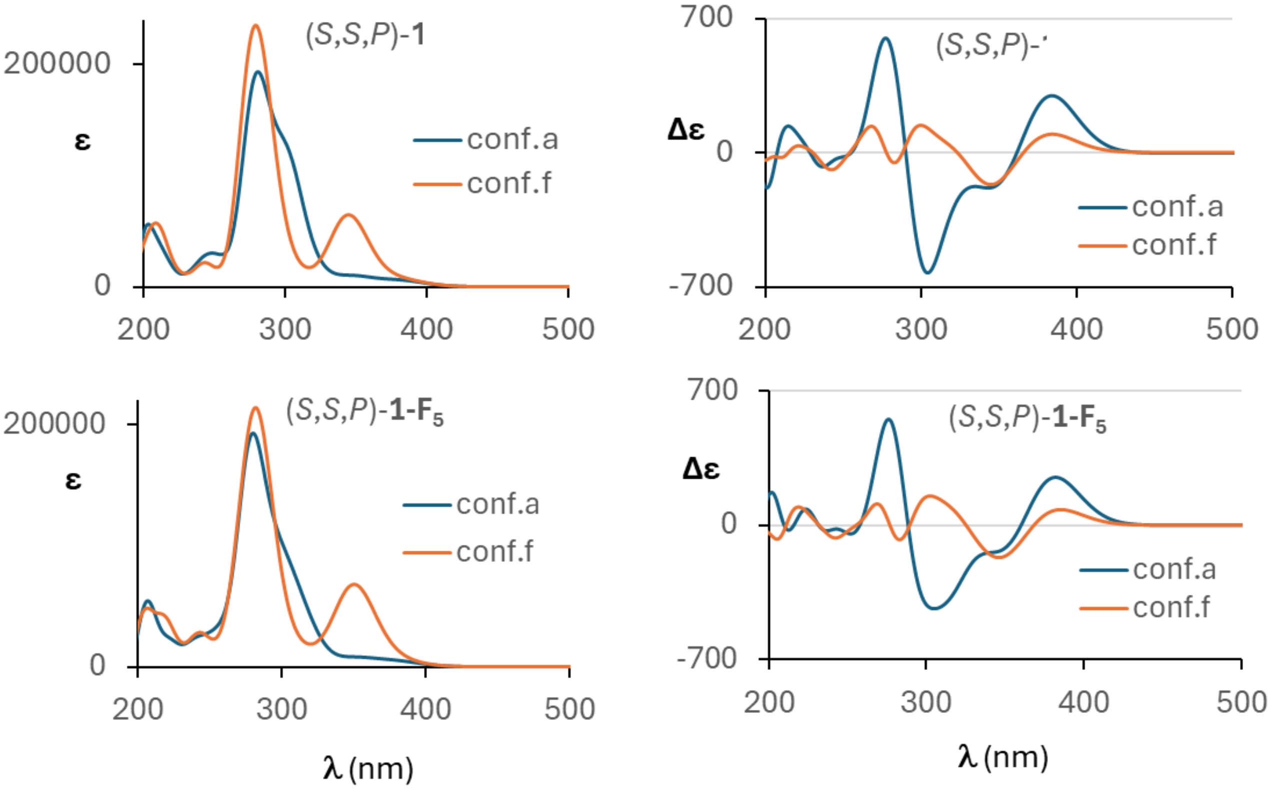

Figure S25. Spectra of the most stable (folded) conformer “a” and that of the partially unfolded structure”f for (*S*,*S*,*P*)-**1** and (*S*,*S*,*P*)-**1-F$_5$**

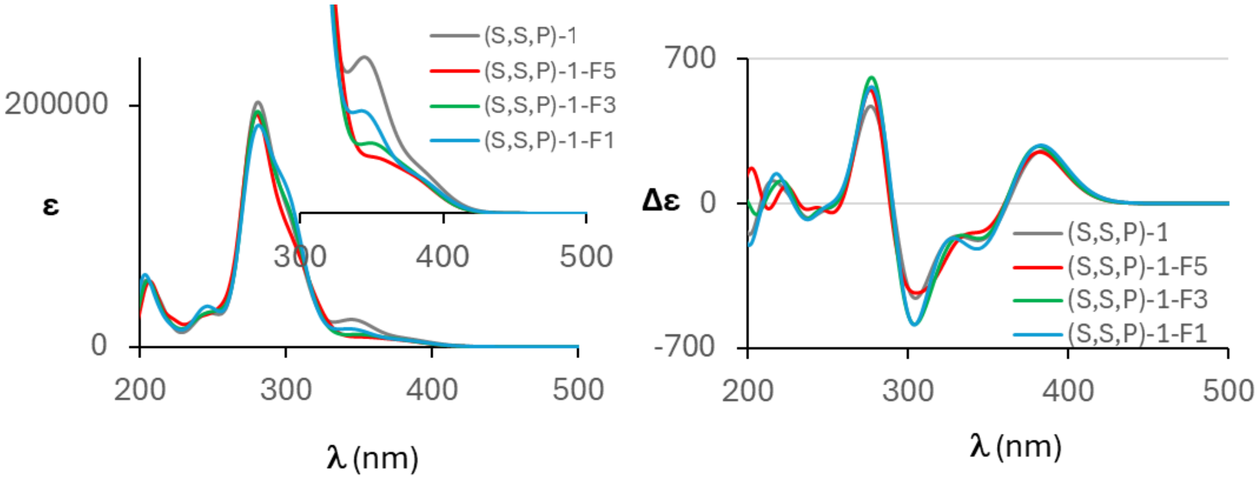

Figure S26. Spectra calculated as average between the one of the folded conformer “a” and that of the partially unfolded structure”f for *(S,S,P)*-**1** to *(S,S,P)*-***1-F$_5$*** average based on populations obtained from energy values reported in **Table S4**.

5.3. Coordinates for the lowest energy structures of compounds (*S*,*S*,*P*)-1-F_n

(*S*,*S*,*P*)-1

C	6.59520	-2.61140	-1.37510
C	6.08340	-1.46220	-0.76550
C	4.69590	-1.17320	-0.79420
C	3.81480	-2.07350	-1.48150
C	4.35710	-3.23620	-2.08200
C	5.72780	-3.50440	-2.03070
C	4.22460	-0.01210	-0.12220
C	2.42230	-1.81600	-1.56050
C	3.92560	1.01270	0.49920
C	3.76060	2.25640	1.16830
C	4.86780	3.14190	1.21110
C	4.77930	4.38750	1.83730
C	3.57170	4.78420	2.43970
C	2.46680	3.92950	2.41410
C	2.53180	2.65800	1.78830
C	1.36940	1.84170	1.79730
C	0.29390	1.24020	1.87310
C	1.20810	-1.60910	-1.63510
C	-0.18930	-1.37330	-1.64980
C	-0.98330	0.62610	1.94240
C	-1.14610	-0.80060	1.88980
C	-2.44290	-1.35020	1.93070
C	-3.57850	-0.52750	2.03490
C	-3.42530	0.87370	2.10320
C	-2.14270	1.42750	2.05620
C	-1.10510	-2.44850	-1.62890
C	-2.48520	-2.23730	-1.56460
C	-2.98420	-0.91710	-1.51260
C	-2.09790	0.17310	-1.56430
C	-0.70730	-0.03140	-1.63810
O	-4.30810	-0.59680	-1.41290
O	-4.77850	-1.19320	2.07740
C	-5.99570	-0.42870	2.00650
C	-5.22700	-1.64570	-1.06510
C	-6.47740	-1.02780	-0.46410
C	-6.23040	0.06560	0.58430
O	-7.44810	0.80960	0.53920
C	-7.84740	0.82660	-0.84760
O	-7.24980	-0.34820	-1.45850
C	-7.29510	2.06090	-1.55820
C	-9.36450	0.71880	-0.89440
H	7.67460	-2.81510	-1.33530
H	6.75110	-0.76600	-0.23950
H	3.67290	-3.92620	-2.59420
H	6.12360	-4.41350	-2.50490
H	5.80220	2.82770	0.72680

H	5.65250	5.05490	1.84960
H	3.49220	5.76360	2.93250
H	1.51990	4.22960	2.88350
H	-2.57800	-2.43840	1.88350
H	-4.29080	1.54170	2.18950
H	-2.02490	2.51870	2.09900
H	-0.71300	-3.47440	-1.64750
H	-3.16150	-3.10040	-1.55030
H	-2.50410	1.19210	-1.52850
H	-6.79450	-1.13100	2.31030
H	-5.98720	0.41870	2.72320
H	-4.77430	-2.31500	-0.30450
H	-5.48720	-2.24360	-1.96480
H	-7.07200	-1.86020	-0.01340
H	-5.36960	0.68960	0.25640
H	-7.56120	2.03140	-2.63260
H	-7.71340	2.98450	-1.11350
H	-6.19110	2.08380	-1.47930
H	-9.71230	0.68720	-1.94430
H	-9.68790	-0.20400	-0.37670
H	-9.82860	1.59160	-0.39550
C	-0.00730	-1.64370	1.79810
C	1.00290	-2.34540	1.71370
C	2.17710	-3.13780	1.58410
C	2.11460	-4.42980	1.00120
H	1.14260	-4.81890	0.66750
H	3.22220	-6.18130	0.37540
C	3.28000	-5.18580	0.83880
C	4.52140	-4.67480	1.25680
C	4.59100	-3.40110	1.84380
H	5.56080	-2.99410	2.16290
C	3.43240	-2.63330	2.00820
H	3.48360	-1.62740	2.44550
C	0.19560	1.06330	-1.69600
C	1.03560	1.96380	-1.75440
C	2.05080	2.95930	-1.80130
C	3.30870	2.65860	-2.38510
H	3.47350	1.65920	-2.80960
H	5.29750	3.37370	-2.85230
C	4.32510	3.61970	-2.40160
C	4.11130	4.88790	-1.83550
C	2.86870	5.19470	-1.25500
H	2.70140	6.18200	-0.80150
C	1.84300	4.24380	-1.23810
H	0.87460	4.47310	-0.77280
H	4.91620	5.63690	-1.84070
H	5.43660	-5.26720	1.11630

(*S*,*S*,*P*)-1-F₁

C	6.33070	-2.63880	-1.45660
C	5.84060	-1.48270	-0.84270
C	4.45770	-1.16950	-0.86410
C	3.55920	-2.04900	-1.55510
C	4.08090	-3.21830	-2.16320
C	5.44600	-3.51350	-2.11350
C	4.01120	-0.01510	-0.16430
C	2.16850	-1.77750	-1.62430
C	3.74910	0.99610	0.49520
C	3.61670	2.21630	1.21250
C	4.73870	3.08250	1.27240
C	4.67780	4.30650	1.94330
C	3.48510	4.69660	2.57910
C	2.36670	3.85970	2.53890
C	2.40190	2.61350	1.86220
C	1.22660	1.81560	1.84300
C	0.14630	1.21910	1.88490
C	0.95300	-1.57440	-1.69260
C	-0.44730	-1.35230	-1.70060
C	-1.13470	0.60970	1.93200
C	-1.30110	-0.81610	1.86400
C	-2.59850	-1.36390	1.90180
C	-3.73270	-0.54070	2.01640
C	-3.57750	0.86010	2.08830
C	-2.29350	1.41200	2.04650
C	-1.35100	-2.43830	-1.69040
C	-2.73350	-2.24440	-1.61740
C	-3.24750	-0.93080	-1.54850
C	-2.37420	0.16990	-1.59040
C	-0.98170	-0.01710	-1.66960
O	-4.57420	-0.62580	-1.44120
O	-4.93120	-1.20780	2.06720
C	-6.15130	-0.44580	2.03140
C	-5.47640	-1.67700	-1.05800
C	-6.70820	-1.05910	-0.41900
C	-6.42920	0.04120	0.61470
O	-7.64770	0.78500	0.60250
C	-8.09020	0.79290	-0.77110
O	-7.51500	-0.38800	-1.39140
C	-7.55760	2.02050	-1.50780
C	-9.60820	0.68840	-0.76990
H	7.40540	-2.86460	-1.41540
H	6.52130	-0.80230	-0.31270
H	3.38350	-3.89370	-2.67680
H	5.82310	-4.43140	-2.58550
H	5.66180	2.77180	0.76440
H	5.55890	4.96290	1.96130

H	3.42710	5.65830	3.10810
H	1.43120	4.15710	3.03200
H	-2.73520	-2.45150	1.84750
H	-4.44190	1.52930	2.17630
H	-2.17410	2.50270	2.09690
H	-0.94710	-3.45930	-1.72420
H	-3.40030	-3.11510	-1.61100
H	-2.79210	1.18350	-1.53930
H	-6.93940	-1.14800	2.36240
H	-6.12200	0.40470	2.74380
H	-4.99540	-2.34060	-0.31000
H	-5.76460	-2.27990	-1.94570
H	-7.28520	-1.89030	0.05600
H	-5.57840	0.66320	0.25820
H	-7.85920	1.98480	-2.57260
H	-7.95800	2.94850	-1.05580
H	-6.45160	2.04000	-1.46570
H	-9.98830	0.64860	-1.80840
H	-9.91730	-0.22910	-0.23450
H	-10.05480	1.56670	-0.26470
C	-0.16530	-1.66160	1.76120
C	0.84100	-2.36610	1.65870
C	2.01230	-3.15320	1.48820
C	1.94800	-4.40850	0.83000
H	0.97570	-4.78450	0.48430
H	3.08030	-6.11090	0.06620
C	3.10940	-5.14820	0.59370
C	4.33720	-4.63200	1.02580
C	4.43530	-3.41210	1.70160
H	5.41960	-3.04360	2.01800
C	3.27110	-2.67220	1.92930
H	3.32770	-1.69730	2.43040
C	-0.09450	1.09060	-1.70040
C	0.73640	2.00110	-1.71440
C	1.74290	3.00400	-1.67630
C	3.02100	2.75110	-2.23900
H	3.20620	1.79170	-2.73900
H	5.04380	3.50870	-2.54920
C	4.04450	3.69700	-2.13490
C	3.78760	4.89510	-1.45950
C	2.53440	5.18830	-0.91070
H	2.38100	6.13880	-0.38350
C	1.51340	4.24140	-1.02290
H	0.52960	4.43890	-0.57660
F	4.79120	5.79930	-1.31900
F	5.47020	-5.33410	0.76780

(*S*,*S*,*P*)-1-F$_3$

C	5.97780	-2.68890	-1.60910
C	5.50680	-1.55450	-0.94260
C	4.12950	-1.21490	-0.95530
C	3.21680	-2.04930	-1.68230
C	3.72020	-3.19610	-2.34710
C	5.08050	-3.51460	-2.31150
C	3.70360	-0.07720	-0.21580
C	1.82710	-1.76510	-1.72520
C	3.45720	0.91820	0.47300
C	3.34380	2.12580	1.21500
C	4.48020	2.97260	1.29200
C	4.43250	4.19330	1.97070
C	3.23980	4.59870	2.59750
C	2.11030	3.77790	2.54600
C	2.13180	2.53430	1.86340
C	0.94600	1.75230	1.83200
C	-0.13890	1.16370	1.86690
C	0.60930	-1.56940	-1.76980
C	-0.79370	-1.36480	-1.75680
C	-1.41550	0.54510	1.90970
C	-1.56570	-0.88180	1.82640
C	-2.85360	-1.44900	1.86360
C	-3.99770	-0.64160	1.99640
C	-3.85960	0.76050	2.08090
C	-2.58370	1.33100	2.03710
C	-1.68370	-2.46230	-1.75960
C	-3.06770	-2.28740	-1.67080
C	-3.59900	-0.98250	-1.57280
C	-2.74010	0.13010	-1.59950
C	-1.34660	-0.03900	-1.69290
O	-4.92760	-0.69670	-1.44920
O	-5.18550	-1.32490	2.05090
C	-6.41690	-0.57960	2.05930
C	-5.81280	-1.76190	-1.06230
C	-7.03330	-1.16320	-0.38430
C	-6.73870	-0.07540	0.65810
O	-7.96550	0.65330	0.69050
C	-8.44870	0.67430	-0.66980
O	-7.86720	-0.48340	-1.32700
C	-7.96290	1.92460	-1.40020
C	-9.96350	0.53800	-0.62580
H	7.04700	-2.93900	-1.57050
H	6.19600	-0.91660	-0.37320
H	3.01220	-3.83880	-2.88770
H	5.44280	-4.41580	-2.82520
H	5.39840	2.65720	0.77780
H	5.32190	4.83750	1.99700

H	3.18930	5.56220	3.12350
H	1.17500	4.08860	3.03110
H	-2.97610	-2.53760	1.79890
H	-4.73230	1.41700	2.18120
H	-2.47920	2.42270	2.09870
H	-1.26770	-3.47730	-1.81830
H	-3.72330	-3.16680	-1.67620
H	-3.16980	1.13710	-1.52390
H	-7.18600	-1.29790	2.40010
H	-6.37940	0.26000	2.78380
H	-5.30910	-2.43370	-0.33710
H	-6.11710	-2.35160	-1.95320
H	-7.59240	-2.00570	0.09240
H	-5.90520	0.56210	0.28760
H	-8.29170	1.89880	-2.45720
H	-8.37160	2.83610	-0.92290
H	-6.85680	1.96840	-1.38610
H	-10.37380	0.51020	-1.65300
H	-10.23790	-0.39570	-0.09950
H	-10.41240	1.39700	-0.09020
C	-0.41310	-1.70300	1.71430
C	0.62170	-2.36310	1.60900
C	1.84050	-3.07220	1.43680
C	1.86660	-4.28250	0.70040
H	0.94890	-4.71060	0.27970
F	3.14910	-6.03530	-0.27820
C	3.08700	-4.90650	0.46210
C	4.28940	-4.37270	0.95170
C	4.24710	-3.18480	1.69530
F	5.41500	-2.66590	2.13430
C	3.04520	-2.52930	1.94670
H	3.05140	-1.58230	2.49850
C	-0.47550	1.08160	-1.69730
C	0.33500	2.00910	-1.67650
C	1.31850	3.02770	-1.57060
C	2.61900	2.80270	-2.08700
H	2.86690	1.87210	-2.61040
F	4.87190	3.54460	-2.30610
C	3.60940	3.75570	-1.87210
C	3.34550	4.93550	-1.16070
C	2.04860	5.15660	-0.67300
F	1.81830	6.28630	0.03120
C	1.03270	4.22730	-0.87350
H	0.03910	4.41320	-0.44870
F	4.32760	5.82330	-0.91690
F	5.46330	-4.97320	0.68230

(*S*,*S*,*P*)-1-F$_5$

C	5.82510	-2.65610	-1.77320
C	5.35460	-1.54560	-1.06760
C	3.97280	-1.22300	-1.04220
C	3.05630	-2.05160	-1.77030
C	3.55950	-3.17190	-2.47940
C	4.92380	-3.47400	-2.48010
C	3.55400	-0.11270	-0.25950
C	1.66070	-1.79530	-1.76270
C	3.32890	0.85580	0.47270
C	3.25420	2.04170	1.25300
C	4.42450	2.83690	1.36550
C	4.41660	4.04770	2.06330
C	3.23090	4.49590	2.67410
C	2.06840	3.72520	2.59030
C	2.05070	2.49100	1.89010
C	0.83360	1.76140	1.83070
C	-0.28000	1.22990	1.85370
C	0.43800	-1.63180	-1.75420
C	-0.96490	-1.43340	-1.71110
C	-1.56610	0.63350	1.88860
C	-1.72320	-0.79410	1.83700
C	-3.01020	-1.35890	1.88690
C	-4.15150	-0.54320	1.99590
C	-4.00860	0.86060	2.03480
C	-2.73100	1.42750	1.98220
C	-1.85520	-2.52870	-1.66660
C	-3.23890	-2.34560	-1.58150
C	-3.76770	-1.03670	-1.53250
C	-2.90610	0.07310	-1.58680
C	-1.51460	-0.10560	-1.67340
O	-5.09510	-0.74180	-1.42700
O	-5.34040	-1.22100	2.07290
C	-6.57120	-0.47420	2.06470
C	-5.98900	-1.78550	-1.00340
C	-7.20460	-1.15160	-0.34930
C	-6.90410	-0.02420	0.64800
O	-8.13260	0.70220	0.66100
C	-8.63040	0.66550	-0.69460
O	-8.03220	-0.50210	-1.31820
C	-8.17660	1.89860	-1.47250
C	-10.14200	0.50230	-0.62820
H	6.89790	-2.89320	-1.76310
H	6.04760	-0.91480	-0.49490
H	2.84700	-3.81060	-3.01850
H	5.28650	-4.35530	-3.02690
H	5.33780	2.49050	0.86250
H	5.33310	4.65130	2.11790

H	3.21180	5.45320	3.21300
H	1.13700	4.06980	3.05910
H	-3.13560	-2.44850	1.84990
H	-4.88050	1.52200	2.10790
H	-2.62190	2.52010	2.00740
H	-1.43830	-3.54450	-1.67970
H	-3.89710	-3.22250	-1.55170
H	-3.33230	1.08300	-1.53540
H	-7.33770	-1.17850	2.43910
H	-6.52600	0.39180	2.75680
H	-5.49160	-2.43430	-0.25350
H	-6.29710	-2.40430	-1.87290
H	-7.76980	-1.96990	0.16150
H	-6.07480	0.60030	0.24670
H	-8.51060	1.82790	-2.52580
H	-8.60310	2.81730	-1.02550
H	-7.07180	1.96790	-1.46600
H	-10.56370	0.43590	-1.64910
H	-10.39300	-0.42000	-0.07080
H	-10.60080	1.36830	-0.11280
C	-0.56500	-1.60720	1.73470
C	0.49240	-2.22820	1.62950
C	1.72750	-2.89160	1.45160
C	1.83080	-4.05530	0.65560
F	0.72770	-4.56620	0.07700
F	3.14890	-5.74150	-0.39000
C	3.06370	-4.66390	0.41090
C	4.22920	-4.11980	0.97140
C	4.15730	-2.97030	1.77110
F	5.28570	-2.43300	2.26870
C	2.91810	-2.37050	2.01080
F	2.86640	-1.26230	2.76870
C	-0.63020	1.00340	-1.68220
C	0.20890	1.90310	-1.64780
C	1.23260	2.87040	-1.53400
C	2.51420	2.63430	-2.08660
F	2.73820	1.51100	-2.78880
F	4.79330	3.26360	-2.37890
C	3.56840	3.52930	-1.88980
C	3.35680	4.69620	-1.14100
C	2.09270	4.96720	-0.59710
F	1.90770	6.07440	0.14420
C	1.04790	4.06160	-0.79540
F	-0.14260	4.31060	-0.22110
F	4.37470	5.54230	-0.91790
F	5.41940	-4.68440	0.71480

5.4. Coordinates of the first (in energy) partially folded structures of (*S*,*S*,*P*)-1-F_n

Partially unfolded (*S*,*S*,*P*)-1

C	3.31360	7.14240	-0.39810
C	3.77560	5.84050	-0.60700
C	2.95230	4.71470	-0.34410
C	1.62710	4.92870	0.16210
C	1.18050	6.26210	0.35830
C	2.00770	7.35370	0.08240
C	3.47640	3.42000	-0.60970
C	0.73990	3.86650	0.48690
C	4.04800	2.36720	-0.90940
C	4.77530	1.19440	-1.24790
C	6.19170	1.22340	-1.24280
C	6.93660	0.08090	-1.55140
C	6.27960	-1.11930	-1.88070
C	4.88240	-1.16930	-1.90210
C	4.10520	-0.02730	-1.58800
C	2.68940	-0.10120	-1.60510
C	1.45960	-0.19760	-1.61330
C	-0.12920	3.06080	0.83670
C	-1.19580	2.20490	1.21700
C	0.05180	-0.34970	-1.58010
C	-0.52990	-1.60860	-1.21620
C	-1.93610	-1.74010	-1.14290
C	-2.76540	-0.64010	-1.41600
C	-2.19630	0.59520	-1.80000
C	-0.81350	0.73360	-1.87390
C	-2.52450	2.68710	1.18160
C	-3.61590	1.87690	1.50720
C	-3.39530	0.53300	1.87690
C	-2.08280	0.03150	1.93990
C	-0.97610	0.84550	1.62810
O	-4.37750	-0.36630	2.20650
O	-4.12700	-0.66390	-1.32470
C	-4.73320	-1.77650	-0.64450
C	-5.75440	0.01940	2.04320
C	-6.09630	-1.35750	-0.12360
H	3.97140	7.99610	-0.61370
H	4.79010	5.66460	-0.98990
H	0.16300	6.41810	0.74200
H	1.63500	8.37460	0.24620
H	6.69590	2.16380	-0.98110
H	8.03460	0.12520	-1.53560
H	6.86060	-2.01950	-2.12600
H	4.36150	-2.09700	-2.17560
H	-2.35240	-2.71400	-0.86080
H	-2.86340	1.44310	-2.00420

H	-0.37150	1.70280	-2.13970
H	-2.69820	3.72790	0.87660
H	-4.62630	2.30060	1.46060
H	-1.92650	-1.01190	2.24220
H	-4.83280	-2.64110	-1.33510
H	-4.11160	-2.07740	0.22370
H	-5.95390	1.01200	2.49820
H	-6.33350	-0.74010	2.60130
C	0.29810	-2.71870	-0.90080
C	1.01920	-3.67860	-0.61620
C	1.86790	-4.77030	-0.27870
C	3.27880	-4.62330	-0.33190
H	3.70370	-3.65700	-0.63360
H	5.20520	-5.56260	-0.03120
C	4.11400	-5.69210	0.01230
C	3.56470	-6.92260	0.41240
C	2.16860	-7.07900	0.46690
H	1.73420	-8.03930	0.77990
C	1.32320	-6.01690	0.12610
H	0.23170	-6.13500	0.17030
C	0.34490	0.33610	1.72830
C	1.50470	-0.07110	1.82210
C	2.85270	-0.51210	1.92850
C	3.16640	-1.89470	1.97090
H	2.35370	-2.63110	1.91170
H	4.72920	-3.38500	2.09450
C	4.49920	-2.31020	2.06990
C	5.53690	-1.36340	2.12620
C	5.23450	0.00810	2.08290
H	6.04290	0.75240	2.11400
C	3.90600	0.43620	1.98620
H	3.66230	1.50570	1.93700
C	-6.14860	0.00910	0.57070
H	-5.51350	0.72300	0.00090
H	-6.44040	-2.15530	0.58010
H	6.58270	-1.69530	2.19630
H	4.22420	-7.75980	0.68190
O	-7.53040	0.35120	0.45950
O	-7.02980	-1.19590	-1.19520
C	-7.96830	-0.15330	-0.82250
C	-9.35920	-0.74340	-0.63600
H	-10.06750	0.03840	-0.29920
H	-9.72670	-1.16450	-1.59110
H	-9.32510	-1.54590	0.12510
C	-7.89880	0.94010	-1.88550
H	-8.17150	0.52650	-2.87580
H	-8.59680	1.76360	-1.64010
H	-6.86890	1.34090	-1.95040

Partially unfolded (*S*,*S*,*P*)-1-F_1

C	2.14970	7.58840	0.41010
C	2.76400	6.41180	-0.02500
C	2.09160	5.16310	0.03530
C	0.76400	5.11490	0.57490
C	0.16010	6.32740	1.00080
C	0.83840	7.54600	0.91980
C	2.76310	4.01490	-0.46570
C	0.01840	3.91220	0.72360
C	3.44900	3.12010	-0.96940
C	4.27890	2.12090	-1.54510
C	5.68430	2.30160	-1.58000
C	6.52140	1.32270	-2.12520
C	5.96980	0.14440	-2.66240
C	4.58500	-0.05200	-2.64570
C	3.71690	0.91510	-2.08230
C	2.32020	0.67250	-2.01530
C	1.11770	0.41290	-1.92060
C	-0.75880	2.98500	0.97620
C	-1.74690	2.01440	1.29050
C	-0.24400	0.04160	-1.78580
C	-0.58210	-1.28810	-1.36660
C	-1.93900	-1.65530	-1.21850
C	-2.95580	-0.71880	-1.46940
C	-2.62610	0.58910	-1.89100
C	-1.29240	0.95860	-2.04050
C	-3.11290	2.38090	1.24470
C	-4.13520	1.48240	1.56110
C	-3.80440	0.16350	1.93960
C	-2.45570	-0.22620	2.00260
C	-1.41750	0.67440	1.68680
O	-4.70740	-0.81360	2.27570
O	-4.28850	-0.97330	-1.32400
C	-4.67590	-2.17190	-0.63160
C	-6.10620	-0.58010	2.03070
C	-6.10040	-2.01020	-0.12550
H	2.69170	8.54240	0.34850
H	3.78330	6.43420	-0.43410
H	-0.85920	6.28460	1.40820
H	0.34540	8.46730	1.26080
H	6.10540	3.22610	-1.16190
H	7.60890	1.47990	-2.13570
H	6.62330	-0.62350	-3.09960
H	4.14590	-0.96180	-3.07730
H	-2.16960	-2.67740	-0.89680
H	-3.43780	1.30600	-2.07220
H	-1.03640	1.98180	-2.34560
H	-3.37070	3.40610	0.94660

H	-5.17750	1.81950	1.50860
H	-2.21360	-1.25380	2.30290
H	-4.60740	-3.04800	-1.31130
H	-4.01180	-2.34060	0.24140
H	-6.43690	0.39190	2.45270
H	-6.63060	-1.38990	2.57150
C	0.45380	-2.21160	-1.06590
C	1.39810	-2.95280	-0.78240
C	2.52290	-3.75220	-0.43850
C	3.83230	-3.30620	-0.75480
H	3.96210	-2.34480	-1.26720
H	5.97030	-3.71110	-0.61130
C	4.95220	-4.05870	-0.39240
C	4.76080	-5.26360	0.29300
C	3.48500	-5.73740	0.62190
H	3.38090	-6.68810	1.16150
C	2.36800	-4.98080	0.25350
H	1.35920	-5.33300	0.50770
C	-0.06710	0.24650	1.76850
C	1.09600	-0.15620	1.84190
C	2.44290	-0.60770	1.88410
C	2.78050	-1.84830	2.48410
H	1.99030	-2.46160	2.93730
H	4.37770	-3.26760	2.92600
C	4.10220	-2.30120	2.48380
C	5.08310	-1.51640	1.86730
C	4.79030	-0.28350	1.27390
H	5.58750	0.29750	0.79030
C	3.46920	0.17280	1.29410
H	3.20930	1.13540	0.83600
C	-6.40810	-0.65810	0.53880
H	-5.85570	0.14190	-0.00090
H	-6.28990	-2.84370	0.59380
F	6.35970	-1.98080	1.82280
F	5.84560	-5.99430	0.65470
O	-7.81560	-0.52840	0.33960
O	-7.04350	-2.07040	-1.20110
C	-8.07430	-1.07400	-0.96900
C	-9.43670	-1.74910	-0.93100
H	-10.23090	-1.00450	-0.72910
H	-9.64780	-2.23400	-1.90280
H	-9.44910	-2.51470	-0.13260
C	-7.95170	0.00290	-2.04690
H	-8.11310	-0.44320	-3.04730
H	-8.70360	0.79980	-1.88730
H	-6.93750	0.44610	-2.03140

Partially unfolded (*S*,*S*,*P*)-1-F_3

C	1.27560	7.85890	0.68050
C	1.94380	6.74680	0.16260
C	1.34090	5.46190	0.15840
C	0.03000	5.30840	0.71790
C	-0.63010	6.45680	1.22850
C	-0.02020	7.71360	1.21020
C	2.05880	4.38430	-0.42770
C	-0.64030	4.05670	0.80510
C	2.77630	3.56480	-1.00960
C	3.63840	2.66100	-1.68650
C	5.02530	2.93570	-1.77290
C	5.89410	2.05110	-2.41840
C	5.39310	0.87670	-3.00920
C	4.02490	0.59180	-2.94910
C	3.12720	1.45950	-2.27980
C	1.75280	1.12050	-2.17160
C	0.57620	0.76790	-2.05600
C	-1.34470	3.06270	1.01150
C	-2.24400	2.00020	1.29180
C	-0.74670	0.28110	-1.90000
C	-0.96160	-1.08620	-1.52210
C	-2.27650	-1.57670	-1.35750
C	-3.37550	-0.72230	-1.54880
C	-3.16800	0.62370	-1.92650
C	-1.87560	1.11280	-2.09450
C	-3.63590	2.25320	1.30720
C	-4.56900	1.25730	1.60800
C	-4.11960	-0.04680	1.90640
C	-2.74220	-0.32680	1.90160
C	-1.79460	0.67330	1.60310
O	-4.92620	-1.10930	2.22490
O	-4.67730	-1.09320	-1.38220
C	-4.94630	-2.34430	-0.72660
C	-6.35020	-0.97470	2.06280
C	-6.35390	-2.30770	-0.15360
H	1.76370	8.84340	0.66770
H	2.95120	6.85030	-0.26290
H	-1.63670	6.33310	1.65080
H	-0.55470	8.58420	1.61570
H	5.40840	3.85380	-1.30750
H	6.96860	2.27710	-2.46180
H	6.07190	0.17930	-3.51950
H	3.62270	-0.31190	-3.42770
H	-2.41020	-2.62560	-1.06920
H	-4.04250	1.27410	-2.06070
H	-1.71700	2.16440	-2.36760
H	-3.98740	3.26720	1.07370

H	-5.63650	1.50910	1.60700
H	-2.40600	-1.34420	2.13960
H	-4.84490	-3.18410	-1.44660
H	-4.23420	-2.49830	0.11030
H	-6.72910	-0.05070	2.54690
H	-6.78180	-1.84570	2.59040
C	0.16240	-1.91660	-1.27210
C	1.19190	-2.54930	-1.02830
C	2.42600	-3.18310	-0.72160
C	3.63680	-2.52130	-1.04330
H	3.63570	-1.54090	-1.53270
F	6.01380	-2.47590	-0.97280
C	4.85080	-3.10500	-0.69520
C	4.90410	-4.33950	-0.03020
C	3.69790	-4.98980	0.27730
F	3.76280	-6.17460	0.92070
C	2.46540	-4.43530	-0.05880
H	1.54470	-4.96770	0.20870
C	-0.41100	0.35780	1.63240
C	0.78310	0.05690	1.67850
C	2.15890	-0.29360	1.72820
C	2.55570	-1.54120	2.27200
H	1.81770	-2.25420	2.65850
F	4.31370	-3.06210	2.79520
C	3.90690	-1.87100	2.30010
C	4.88390	-0.99960	1.79500
C	4.47890	0.23550	1.26390
F	5.43120	1.06160	0.78040
C	3.13610	0.60150	1.22620
H	2.84740	1.57240	0.80520
C	-6.72440	-1.01210	0.58600
H	-6.26200	-0.15040	0.05640
H	-6.45110	-3.18310	0.53390
F	6.18220	-1.35360	1.80030
F	6.08390	-4.88590	0.31240
O	-8.14590	-0.98210	0.46280
O	-7.33870	-2.38650	-1.18930
C	-8.43340	-1.49210	-0.85460
C	-9.73170	-2.28050	-0.77460
H	-10.57010	-1.61540	-0.49050
H	-9.96130	-2.73630	-1.75630
H	-9.63390	-3.08030	-0.01660
C	-8.46090	-0.36380	-1.88520
H	-8.63700	-0.77890	-2.89640
H	-9.26820	0.35680	-1.65090
H	-7.48880	0.16550	-1.89850

Partially unfolded (*S*,*S*,*P*)-1-F_5

C	-0.41140	8.35770	0.28450
C	0.49500	7.38670	-0.14760
C	0.21450	6.00170	-0.01190
C	-1.02110	5.60270	0.59630
C	-1.92750	6.61040	1.02050
C	-1.63110	7.96690	0.86800
C	1.17330	5.07140	-0.49540
C	-1.39910	4.24670	0.80450
C	2.08350	4.38420	-0.96730
C	3.17540	3.63430	-1.47910
C	4.45980	4.22700	-1.55260
C	5.55960	3.50400	-2.02420
C	5.39820	2.17030	-2.44170
C	4.13820	1.56660	-2.39060
C	3.01000	2.27650	-1.91330
C	1.73780	1.64960	-1.85460
C	0.63410	1.10090	-1.79800
C	-1.89640	3.15070	1.08540
C	-2.63990	1.98100	1.39670
C	-0.63290	0.47030	-1.71910
C	-0.74080	-0.92500	-1.41360
C	-2.01250	-1.53360	-1.30130
C	-3.17750	-0.77170	-1.48770
C	-3.07690	0.60080	-1.81080
C	-1.82820	1.20610	-1.91730
C	-4.05370	2.05750	1.39820
C	-4.85970	0.94830	1.66500
C	-4.25740	-0.29770	1.94340
C	-2.85660	-0.39970	1.97350
C	-2.03760	0.71750	1.70740
O	-4.93250	-1.46190	2.20410
O	-4.44410	-1.26210	-1.36880
C	-4.60760	-2.56990	-0.79310
C	-6.34810	-1.51280	1.94630
C	-6.04350	-2.72640	-0.32090
H	-0.16880	9.42280	0.16400
H	1.44680	7.68060	-0.61090
H	-2.87380	6.29670	1.48220
H	-2.35170	8.72370	1.20850
H	4.57710	5.26750	-1.22010
H	6.54860	3.98150	-2.06530
H	6.26040	1.59810	-2.81210
H	4.00240	0.53050	-2.72300
H	-2.05790	-2.60190	-1.06140
H	-4.00010	1.17950	-1.94730
H	-1.75490	2.27870	-2.13920
H	-4.52520	3.02270	1.16950

H	-5.95010	1.06580	1.64730
H	-2.39640	-1.37020	2.19950
H	-4.36350	-3.35270	-1.54260
H	-3.93570	-2.68760	0.08260
H	-6.88210	-0.67360	2.43900
H	-6.68840	-2.45870	2.40730
C	0.42440	-1.70400	-1.19230
C	1.42540	-2.39370	-0.99120
C	2.58050	-3.16930	-0.73980
C	3.87720	-2.66170	-0.99020
F	4.01880	-1.41080	-1.47030
F	6.24480	-2.88630	-0.94740
C	5.02680	-3.40720	-0.72210
C	4.90290	-4.70310	-0.19800
C	3.63210	-5.23670	0.06550
F	3.52140	-6.47500	0.57600
C	2.49080	-4.47360	-0.19840
F	1.28260	-4.99650	0.06990
C	-0.62960	0.55170	1.73770
C	0.58170	0.33250	1.76380
C	1.96880	0.06800	1.73300
C	2.47900	-1.22250	2.00300
F	1.63600	-2.22440	2.31070
F	4.31090	-2.73240	2.18770
C	3.84670	-1.49480	1.92590
C	4.73850	-0.48390	1.53660
C	4.26430	0.81180	1.27640
F	5.12280	1.77940	0.91360
C	2.89840	1.08070	1.39980
F	2.45460	2.32580	1.17210
C	-6.61940	-1.52580	0.44630
H	-6.23900	-0.58870	-0.01630
H	-6.08090	-3.64270	0.31750
F	6.04540	-0.76260	1.41000
F	6.00050	-5.42730	0.06340
O	-8.02300	-1.66750	0.22990
O	-6.93840	-2.86190	-1.42970
C	-8.16390	-2.14470	-1.12370
C	-9.34300	-3.10510	-1.15710
H	-10.27900	-2.57590	-0.89280
H	-9.45560	-3.53460	-2.17050
H	-9.17580	-3.92380	-0.43200
C	-8.29100	-0.97940	-2.10400
H	-8.34700	-1.36170	-3.14160
H	-9.20470	-0.39120	-1.89190
H	-7.40500	-0.31990	-2.03070

6. REFERENCES

[S1] Ortuño, A. M; Reiné, P.; Resa, S.; Álvarez de Cienfuegos, L.; Blanco, V.; Paredes, J. M.; Mota, A. J.; Mazzeo, G.; Abbate, S.; Ugalde, J. M.; Mujica, V.; Longhi, G.; Miguel, D.; Cuerva, J. M. Extended Enantiopure Ortho-Phenylene Ethylene (o-OPE)-based Helical Systems as Scaffolds for Supramolecular Architectures: a Study of Chiroptical Response and its Connection to the CISS Effect. *Org. Chem. Front.* **2021**, *8*, 5071-5086.

[S2] Shida, N.; Owaki, S.; Eguchi, H.; Nishikawa, T.; Tomita, I.; Inagi, S. Bis (Pentafluorophenyl)-*o*-Carborane and its Arylthio Derivatives: Synthesis, Electrochemistry and Optical Properties. *Dalton Trans.* **2020**, *49*, 12985-12989.

[S3] Tahara, K.; Fujita, T.; Sonoda, M.; Shiro, M.; Tobe, Y. Donors and Acceptors Based on Triangular Dehydrobenzo [12] Annulenes: Formation of a Triple-Layered Rosette Structure by a Charge-Transfer Complex. *J. Am. Chem. Soc.* **2008**, *130*, 14339-14345.

[S4] Olmsted, J. Calorimetric Determinations of Absolute Fluorescence Quantum Yields. *J. Phys. Chem.* **1979**, *83*, 2581-2584.

[S5] a) Valeur, B.; Berberan-Santos, M. N. *Molecular Fluorescence. Principles and Applications, 2nd ed.*; Wiley-VCH: Weinheim, 2012. b) Lakowicz, J. *Principles of Fluorescence Spectroscopy, 3rd ed.*; Springer-Verlag: New York, 2006.

[S6] Grimme, S. *J. Chem. Theory Comput.* **2019**, *15*, 2847–2862.

[S7] Grimme, S.; Antony, J.; Ehrlich, S.; Krieg, H. *J. Chem. Phys.* **2010**, *132*, 154104.

[S8] Frisch, M. J.; Trucks, G. W.; Schlegel, H. B.; Scuseria, G. E.; Robb, M. A.; Cheeseman, J. R.; Scalmani, G.; Barone, V.; Petersson, G. A.; Nakatsuji, H.; Li, X.; Caricato, M.; Marenich, A. V.; Bloino, J.; Janesko, B. G.; Gomperts, R.; Mennucci, B.; Hratchian, H. P.; Ortiz, J. V.; Izmaylov, A. F.; Sonnenberg, J. L.; Williams-Young, D.; Ding, F.; Lipparini, F.; Egidi, F.; Goings, J.; Peng, B.; Petrone, A.; Henderson, T.; Ranasinghe, D.; Zakrzewski, V. G.; Gao, J.; Rega, N.; Zheng, G.; Liang, W.; Hada, M.; Ehara, M.; Toyota, K.; Fukuda, R.; Hasegawa, J.; Ishida, M.; Nakajima, T.; Honda, Y.; Kitao, O.; Nakai, H.; Vreven, T.; Throssell, K.; Montgomery, J. A., Jr.; Peralta, J. E.; Ogliaro, F.; Bearpark, M. J.; Heyd, J. J.; Brothers, E. N.; Kudin, K. N.; Staroverov, V. N.; Keith, T. A.; Kobayashi, R.; Normand, J.; Raghavachari, K.; Rendell, A. P.; Burant, J. C.; Iyengar, S. S.; Tomasi, J.; Cossi, M.; Millam, J. M.; Klene, M.; Adamo, C.; Cammi, R.; Ochterski, J. W.; Martin, R. L.; Morokuma, K.; Farkas, O.; Foresman, J. B.; Fox, D. J. Computer code GAUSSIAN16, Revision C.01, Gaussian, Inc. Wallingford, CT.
